# Genome-Wide Linkage-Disequilibrium Mapping to the Candidate Gene Level in Melon (*Cucumis melo*)

**DOI:** 10.1038/s41598-017-09987-4

**Published:** 2017-08-29

**Authors:** Amit Gur, Galil Tzuri, Ayala Meir, Uzi Sa’ar, Vitaly Portnoy, Nurit Katzir, Arthur A. Schaffer, Li Li, Joseph Burger, Yaakov Tadmor

**Affiliations:** 1 Plant Science Institute, Agricultural Research Organization, Newe Ya’ar Research Center, P.O. Box 1021, Ramat Yishay, 3009500 Israel; 2 Plant Science Institute, Agricultural Research Organization, The Volcani Center, P.O. Box 15159, Rishon LeZiyyon, 7505101 Israel; 3 Robert W Holley Center for Agriculture and Health, USDA-ARS, Plant Breeding and Genetics Section, School of Integrative Plant Science, Cornell University, Ithaca, New York, 14853 USA

## Abstract

*Cucumis melo* is highly diverse for fruit traits providing wide breeding and genetic research opportunities, including genome-wide association (GWA) analysis. We used a collection of 177 accessions representing the two *C. melo* subspecies and 11 horticultural groups for detailed characterization of fruit traits variation and evaluation of the potential of GWA for trait mapping in melon. Through genotyping-by-sequencing, 23,931 informative SNPs were selected for genome-wide analyses. We found that linkage-disequilibrium decays at ~100 Kb in this collection and that population structure effect on association results varies between traits. We mapped several monogenic traits to narrow intervals overlapping with known causative genes, demonstrating the potential of diverse collections and GWA for mapping Mendelian traits to a candidate-gene level in melon. We further report on mapping of fruit shape quantitative trait loci (QTLs) and comparison with multiple previous QTL studies. Expansion of sample size and a more balanced representation of taxonomic groups might improve efficiency for simple traits dissection. But, as in other plant species, integrated linkage-association multi-allelic approaches are likely to produce better combination of statistical power, diversity capture and mapping resolution in melon. Our data can be utilized for selection of the most appropriate accessions for such approaches.

## Introduction

Melon (*Cucumis melo* L.: Cucurbitaceae) contains a wealth of phenotypic diversity for multiple attributes, especially for fruit traits such as size, shape, external (rind) and internal (flesh) color, sugar content, acidity, texture and aroma^1^. This wide diversity is the source for ongoing genetic research and breeding aimed at mapping and identifying quantitative trait loci (QTLs) and genes affecting key horticultural and consumers’ preference traits. Numerous genetic studies in recent years focused on fruit quality traits in melon, including fruit size and shape^2–5^, flesh color^6–9^, rind color^10^, netting and sutures^11, 12^, sweetness and aroma^13, 14^, acidity^15^ and ripening behavior^16^. Most of these studies utilized targeted bi-parental populations for the genetic analyses and trait mapping.

Bi-parental linkage mapping was, to a large extent, the default genetic mapping approach for simple and quantitative traits in plant species where generation time is short and the development of segregating populations is feasible. This approach is based on the analysis of the segregation of polymorphism between the parental lines and their progeny. Various linkage population types are commonly used in plant genetics, such as: F_2:3_, recombinant inbred lines (RILs), double-haploids (DH), introgression lines (ILs) or backcross inbred lines (BILs). While such populations display different attributes with respect to time and cost of creation, statistical power and genetic resolution, they all share the advantages and disadvantages of bi-allelic, non-structured designs.

Approximately 15 years ago, association mapping methodology started to become a valid alternative strategy for trait mapping in plants^17, 18^. Among the advantages of association genetics is the use of existing collections, the ability to simultaneously analyze wide phenotypic diversity resulting from multi-allelic genetic variation, and the exploitation of accumulated historical recombination events. A key limiting factor that inhibited the effective implementation of this approach in plant genetics was the inherent effect of population structure on creation of spurious associations. The development and successful application of statistical and computational tools to control for genetic relatedness^19–21^ enhanced the use of this approach in plants. At first, the implementation of association mapping was mostly through the analysis of candidate-genes, due to the insufficient genome-wide marker coverage defined by linkage-disequilibrium (LD) decay profile and genome size^22–24^. However, with the growing application of NGS-based approaches for high-density genotyping, the genome wide association study (GWAS) approach is now extensively used for genome-wide genetic dissection of traits in many crop plants^25, 26^.

Several recent studies in melon have used germplasm collections for characterization of diversity, population structure and for trait mapping. Tomason *et al*. screened 87 melon accessions with 286 simple sequence repeat (SSR) markers to describe population structure and fruit traits variation^27^. Leida *et al*. used a set of 175 melon accessions genotyped with 251 single nucleotide polymorphisms (SNPs; 148 background SNPs and 103 within candidate genes for sugar metabolism and ripening) to describe population structure, LD and candidate gene associations for sugar accumulation and ripening behavior^28^. Two recent studies used genotyping-by-sequencing (GBS) to genotype melon panels and to describe population structure and LD using genome-wide high density markers coverage (13,789 SNPs and 25,422 SNPs)^29, 30^.

In the current study a diverse collection of 177 melon accessions was extensively phenotyped for a number of traits and genotyped with 23,931 SNPs using GBS. The main objective was to describe genetic properties of this diversity panel and to test the actual potential and effectiveness of the GWAS approach for trait mapping in melon. Population structure and genome-wide LD patterns are described. Through GWA analysis, we demonstrate the mapping of several Mendelian traits to a narrow interval overlapping with known causative genes, providing a first proof-of-concept for the potential of association genetics for high resolution mapping to a candidate-gene level in melon.

## Results

### Phenotypic diversity across GWAS panel

**Figure 1:**
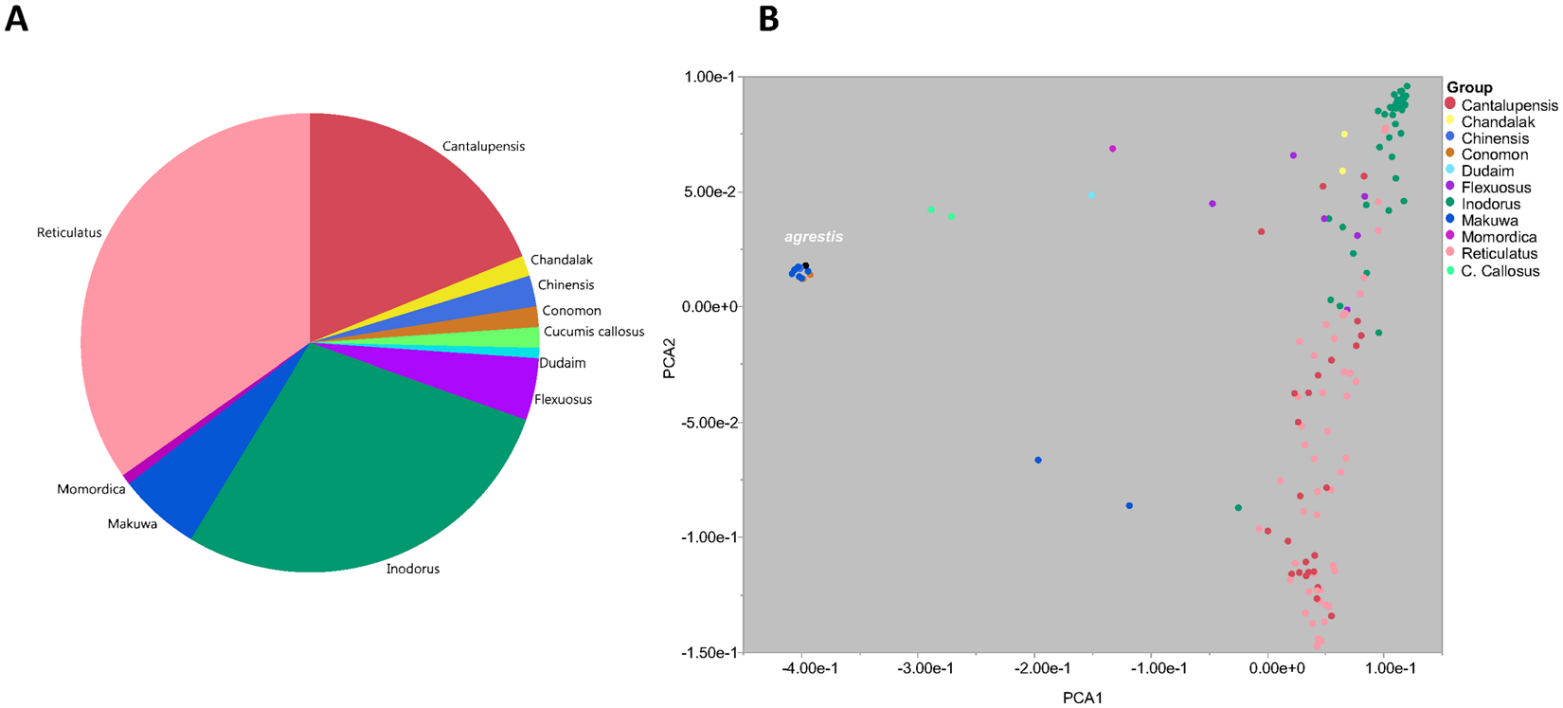
Germplasm composition and structure. (**A**) Pie chart of the frequencies of the different horticultural groups across the melon collection. (**B**) Genetic PCA plot colored by horticultural group.

**Figure 2:**
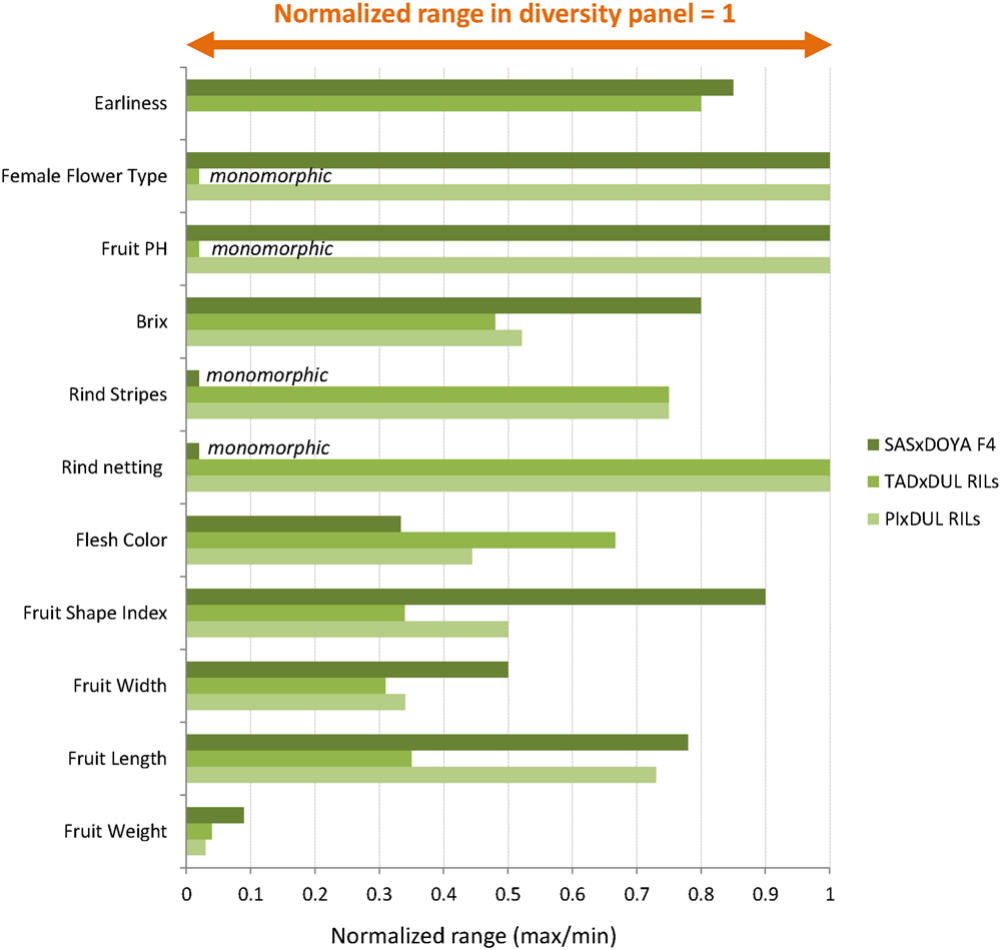
Comparison of normalized phenotypic variation between the GWAS collection and bi-parental melon populations for various traits. Phenotypic range is expressed as the ratio between maximum and minimum entry means for each set where all values are adjusted relative to the range at the GWAS panel (that is therefore normalized to 1).

A diversity panel of 177 inbred melon accessions was used in the current study. This panel represents the two melon subspecies (*melo* and *agrestis*), encompassing 11 horticultural groups (Fig. 1A,B Table S1). The collection was grown in a replicated trial in the open field at Newe Ya’ar in summer 2015 and phenotyped for fruit traits during fruit development and at maturity. Images were taken on developing fruits of all accessions throughout the season from anthesis till harvest. Fifteen mature fruits per accession were harvested from three replicated plots for phenotyping which allowed us to obtain high heritability values for the measured traits. One of the key advantages of using diverse collections compared to bi-parental segregating populations is the extended range of phenotypic variation captured across multiple traits. Figure 2 shows normalized phenotypic ranges (ratio between min and max entry means) in three bi-parental melon populations, relative to the range measured in the GWAS panel, where the ratio is standardized to 1, across numerous selected phenotypic traits (Table S2). These reference experimental bi-parental populations are derived from wide crosses aimed at mapping key fruit quality traits (details at the materials and methods and Figure S1). Range for fruit weight, for example, in the GWAS panel is between 50 g for the smallest accession and 5,000 g for the largest (100-fold difference) while the maximum polymorphism for this trait in the bi-parental populations is 5-fold for the SAS × DOYA cross. For total soluble solids (TSS) the range across the GWAS panel is between 3.5% and 16.2% TSS (4.6-fold) while the ranges within the bi-parental populations are 3-, 2.4- and 1.1-fold. Flesh color is another example where the GWAS panel capture wide phenotypic spectrum in melon (from orange, through white, to green including the different intensities within each color category) and each of the bi-parental crosses captures only a fraction of this spectrum (SAS × DOYA: green, TAD × DUL: green-orange, PI × DUL: orange). The overall extended phenotypic variation captured within the GWAS panel compared to bi-parental populations is evident in our comparison also for time to maturity (i.e. earliness), fruit shape, rind stripes, ripening behavior and many of the other attributes that were measured.

### Genetic variation and genome-wide LD patterns

The collection was genotyped using GBS approach^31^. Over 500,000,000 good barcoded reads were produced and 4,213,896 sequence-tags were extracted with minimum of 3 reads per tag. Fifty-five percent of the tags were uniquely aligned, 2.1% were aligned to more than one locus and 42.9% were unaligned. Such alignment rate for GBS tags in a plant genome can result from several possible factors: (1) Short tag sequence (too short to align significantly), (2) Tag sequence contains highly repetitive DNA, (3) The haplotype is too divergent from the reference to align, (4) Structural variation: the tag sequence is not present in the reference genome individual, or (5) Incomplete reference: the tag sequence is not present in the reference genome.

**Figure 3:**
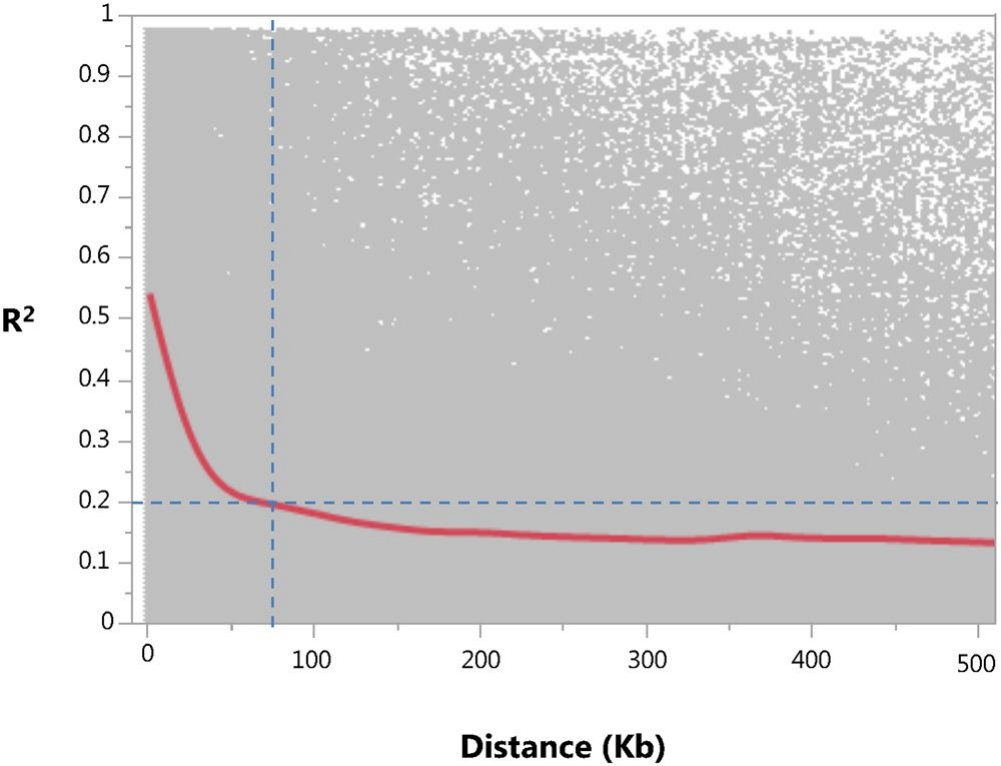
Genome-wide LD decay plot. R2 between intra-chromosomal marker pairs plotted against the physical distance between them.

A total of 99,263 SNPs that were mapped to the melon genome^32^ were identified across the 177 melon accessions. Following further filtration to minor allele frequency (MAF) > 0.05 and maximum of 6% missing data per site, 23,931 informative SNP markers were defined as the genotypic dataset in this study. As expected in GBS, SNP densities fluctuate within and between chromosomes, resulting in differential coverage across the genome (Figure S2) with a genome-wide average density of ~1 SNP/18 Kb. Principal component analysis (PCA) was performed using the whole-genome marker data. Two-dimensional PCA plot using PCA component#1 and PCA component#2 is presented in Fig. 1B where the 11 horticultural groups are color-coded. In general, good consensus is observed between the phenotypically defined groups and the clustering based on genetic relatedness, with an obvious separation between the *melo* and *agrestis* sub-species (also shown as a phylogenetic tree in Figure S3). Also, the *inodorous* group is nicely clustered and separated from the *reticulatus* and *cantalupensis* types that cluster together with wide overlapping genetic variation within them. An admixture-based clustering model implemented in the software STRUCTURE^33^ was also used to infer the genetic structure of the collection. Clear division between the two subspecies was obtained at K = 2 across the whole collection, and K = 7 provided the second best fit for sub-grouping the accessions, in agreement with the taxonomic classification (Figure S4). We then used the genome-wide marker set to characterize patterns of LD across the genome. LD was calculated as R^2^ between SNP pairs and plotted against their physical distance. On a genome-wide average, LD decay in this melon collection within ~100 Kb to a level below R^2^ = 0.2 (Fig. 3). Substantial variation in local LD patterns that exists along and between chromosomes is shown in Figures S2 and S5, where LD is plotted against physical positions or distance. Based on the genome-wide LD decay pattern (~100 Kb) and melon genome size (~450 Mb), the estimated minimal number of markers required for efficient genome-wide scan using a panel similar in composition to the one used here would be ~5,000.

### Whole genome LD mapping

While LD decay analysis provides a general averaged view on potential genetic resolution, we took a direct comparative mapping approach. To test the usefulness of GWAS approach for mapping traits using the melon diversity collection, we phenotyped and performed association mapping for several high heritability simple traits where causative genes are known. The obtained mapping results provide insight into local resolutions that can be achieved using this platform.

### Characterization and mapping of sex determination trait

**Figure 4:**
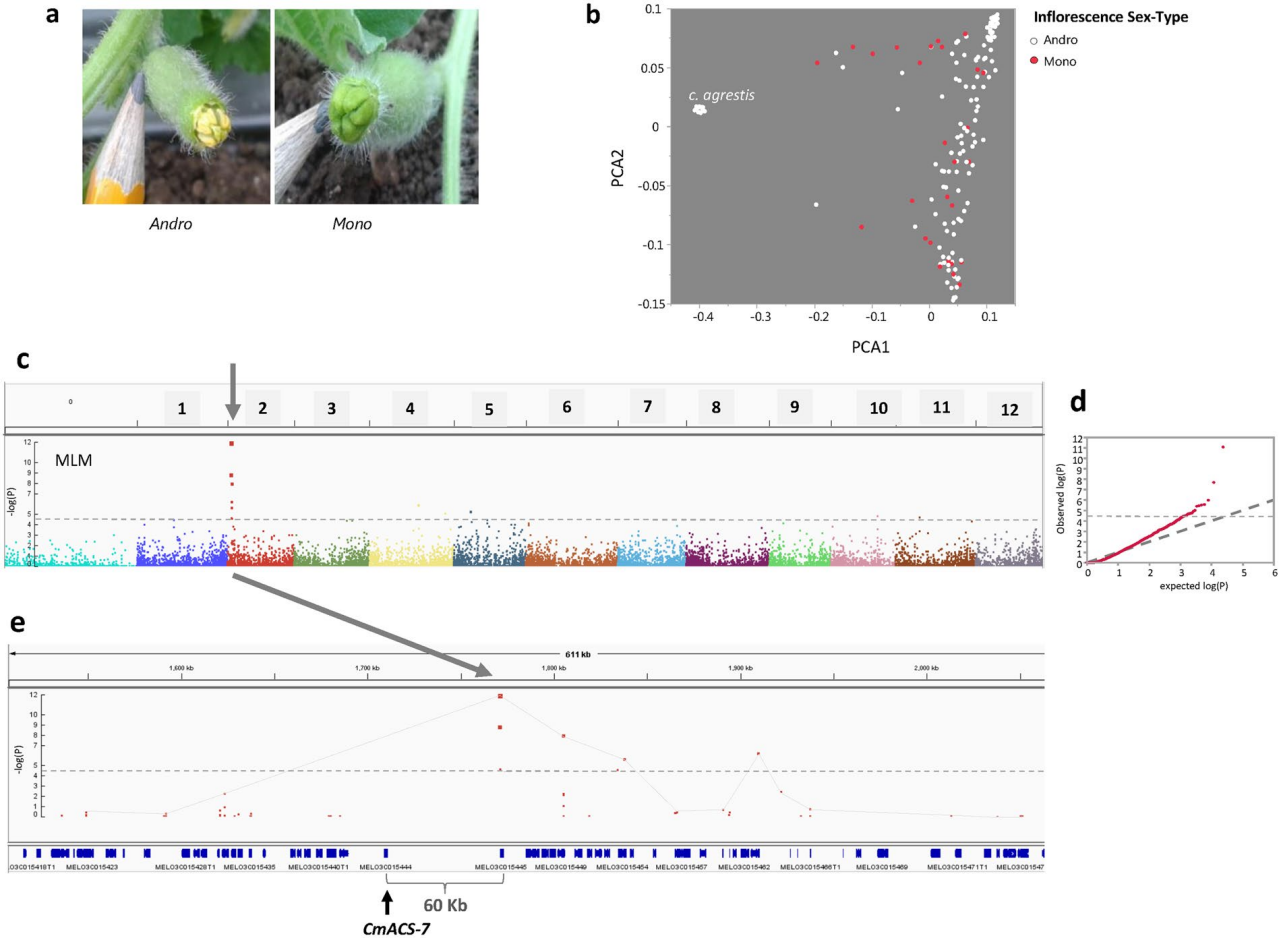
Mapping of flower sex-expression. (**a**) Examples of monoecious (right) and andromonoecious (left) female flowers at anthesis in the diversity panel. (**b**) Female flower types projected on genetic PCA plot. (**c**) Manhattan plot of GWA of female flower type. Associations were tested using mixed-linear model (MLM_Q + K) for controlling population structure and relatedness. Genome-wide significance threshold is adjusted for multiple comparisons at FDR5%. (**d**) Quantile-quantile (Q-Q) plot for distribution of P values at the MLM_Q + K model. The negative logarithm of the observed (y axis) and the expected (x axis) P value is plotted for each SNP (dot). The gray dashed line indicates the null hypothesis. (**e**) Zoom in on chromosome 2 peak where causative gene *CmACS-7* ^35^ (MELO3C015444) is shown.

In melon, most plants are either monoecious or andromonoecious. Monoecious female flowers contain only the female reproductive organs (carpels) and andromonoecious contain both female and male (stamens) organs. This trait was previously mapped to the *a* locus^34^ on chromosome 2 and the causative gene (*CmACS-7*) was subsequently cloned^35^. In the current study, female flowers at anthesis were visually characterized for their sex-expression type (monoecious or andromonoecious, Fig. 4a) across all accessions. Sixteen percent of the accessions in the collection are monoecious and 84% are andromonoecious. Projection of the sex expression phenotypes on the genetic PCA plot provides an informative view on the distribution of the phenotypic classes across the genetic variation and population structure (Fig. 4b). While the main *C. agrestis* cluster in our sample (containing *makuwa*, *chinensis* and *conomon* accessions) is monomorphic, showing only the andromonoecious phenotype, for the rest of our panel the distribution of the phenotypic classes is relatively uniform across the genetic variation and independent of population structure, which is an important attribute for effective association analysis. Using these phenotypes we then performed GWA analysis and found a single significant locus on chromosome 2 associated with this trait (P = 1.4 × 10^−12^ at mixed linear model (MLM) analysis, Fig. 4c,d). Zooming in on the genomic neighborhood of this locus showed that the peak of the trait locus is at position 1,771,409, ~60 Kb and one gene apart from the *CmACS-7* gene, in which we did not have a SNP in our genotyping set (MELO3C015444: Chr2 1,708,995–1,711,002, Fig. 4e). To further validate the peak marker SNP for this trait (SNP1771409), we have genotyped a set of lines from the panel using PCR marker at the *CmACS-7* gene sequence^35^. High LD is found between SNP1771409 and the *CmACS-7* PCR marker (Figure S6). Figure 4e also shows the decline of significance bellow the genome-wide threshold at a less than 100 KB window around the trait-locus peak, supported by the local LD pattern around this locus (Figure S7).

### Characterization and mapping of flesh color

**Figure 5:**
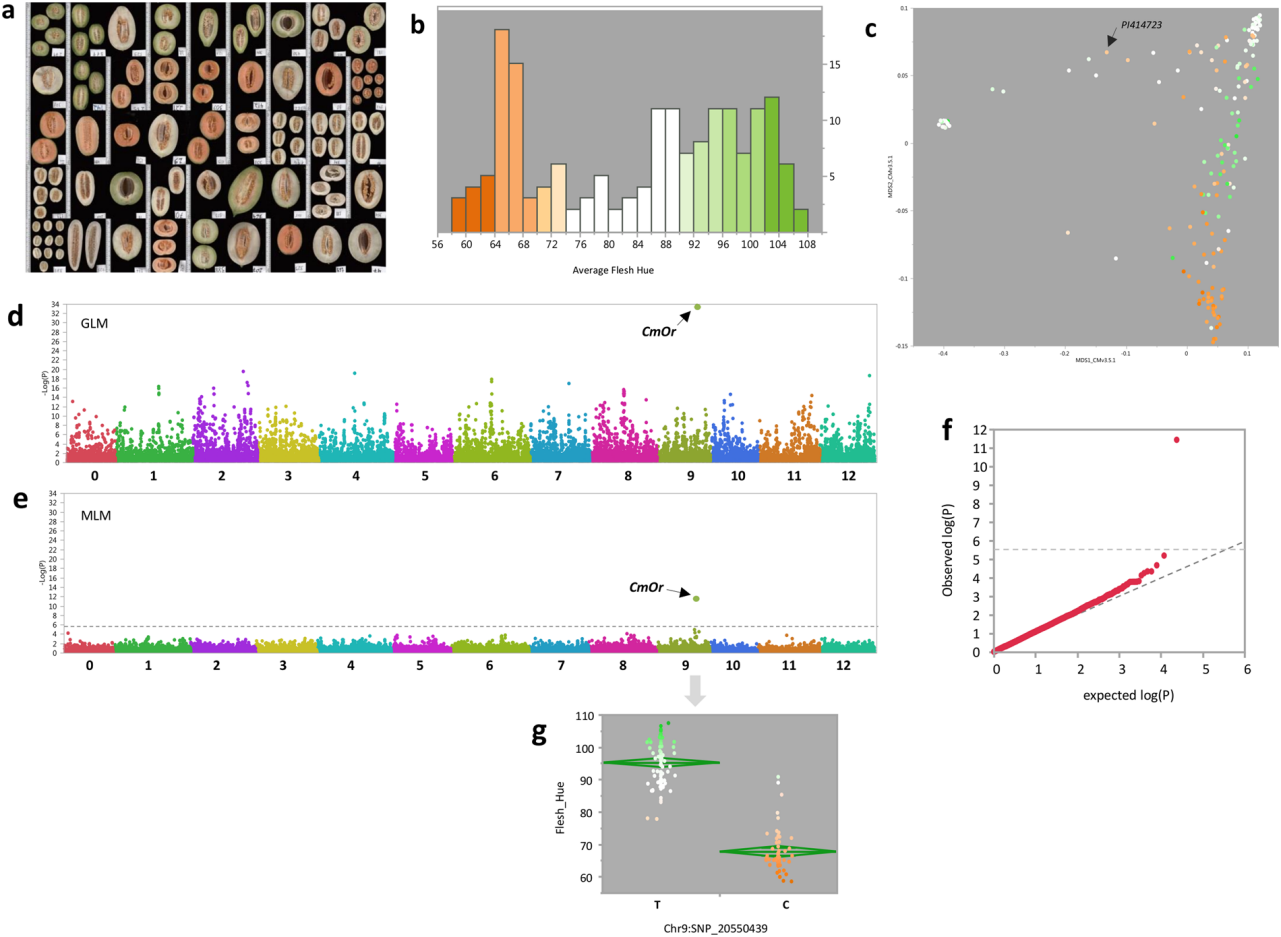
Mapping of flesh color. (**a**) Example of scanned longitudinal sections of melons from the diversity panel. 2,650 fruits were scanned and analyzed using Tomato Analyzer software^36^. (**b**) Frequency distribution of entry mean hue index as measured from fruit scans. Bar colors reflect the visual perception. (**c**) Flesh hue indexes projected on genetic PCA plot. (**d**) Manhattan plot of GWA of flesh color. Associations were tested using naïve General-linear model (GLM) (**e**) Manhattan plot of GWA of flesh color. Associations were tested using mixed-linear model (MLM_Q + K) for controlling population structure and relatedness. Genome-wide significance threshold is adjusted for multiple comparisons at FDR5%. (**f**) Quantile-quantile (Q-Q) plot for distribution of P values at the MLM_Q + K model. The negative logarithm of the observed (y axis) and the expected (x axis) P value is plotted for each SNP (dot). The gray dashed line indicates the null hypothesis. (**g**) Allelic effect plot for the SNP within *CmOr* gene (MELO3C005449). Each point represents accession mean color (hue index) and points are color-coded based on the visual color perception.

Three major flesh color categories are defined in melon; green, white and orange, with β-carotene and chlorophyll being the predominant pigments of the orange and green phenotypes, respectively. The major locus differentiating between orange and non-orange flesh is *gf*, previously mapped to chromosome 9^7^. Recently, the gene *CmOr* was identified as *gf* and accounts for most of the orange/non-orange variation in melon^9^. In order to characterize flesh color variation in the GWAS panel, longitudinal sections of fifteen fruits per accession were scanned and analyzed for multiple characteristics, including flesh color, using the Tomato Analyzer software^36^ (Fig. 5a). Nine of the fruits from each accession were also measured for flesh color using handheld colorimeter. Image-based color values were highly correlated with colorimeter measurements across 1500 fruits that were measured in parallel (R = 0.94, Figure S8). Overall flesh color showed very high broad-sense heritability in our experiment (H^2^ = 0.96, Figures S9 and S10). Flesh color phenotypes distribute uniformly within the GWAS panel with all three color categories represented in a significant proportion (Fig. 5b). Projection of flesh color variation on the genetic PCA plot reveals the strong relationship between this trait and population structure (Fig. 5c). A large proportion of the orange accessions class in the *reticulatus* group and most of the white flesh accessions are in the *inodorous* group. *Agrestis* accessions are mostly white flesh. This non-random distribution and clear dependence between the genetic landscape and phenotypic variation is expected to yield excessive spurious associations if a simple statistical model is used for association analysis. This confounding effect can be controlled through the use of relatedness estimates as cofactor in MLM analysis^21^. We performed GWA analysis using both statistical models. As expected, the generalized linear model (GLM) resulted in high proportion of significant SNP effects across all chromosomes (Fig. 5d). However, a single SNP on chromosome 9 (at position 20,550,439) was noticeable for its strong and highly significant effect (P = 10^−34^). MLM analysis removed most of the significant effects (a mix of mostly spurious and maybe some positive associations) and only the chromosome 9 SNP remained highly significant (P = 3.7 × 10^−12^) and passed the genome-wide threshold (false discovery rate (FDR) 5%, Fig. 5e,f). Zooming in on this SNP reveals that it is located within the causative *CmOr* gene (MELO3C0005449: Chr9 20,548,319–20,555,636). This SNP has MAF of 0.32, it explain ~70% of the flesh color variation across the panel and effectively distinguish between orange and non-orange (Fig. 5g). The closest SNPs around this trait-peak marker are 40 Kb apart. This is a cluster of 3 SNPs in LD with each other that show P values of ~10^−4^, indicating the decline of LD at less than 100 Kb in this region. To further validate SNP20550439, we have genotyped all the lines in the panel for the polymorphism in the *CmOr* gene, as described by Tzuri *et al*. (2015)^9^, and obtained 99% match in allelic segregation (Figure S11). In consensus with the local LD decay in this region (Figures S2 and S12), this example also demonstrates the decline of significance within a relatively short interval around the peak, producing a manageable window of candidates that include the causative gene.

### Characterization and mapping of yellow rind color

**Figure 6:**
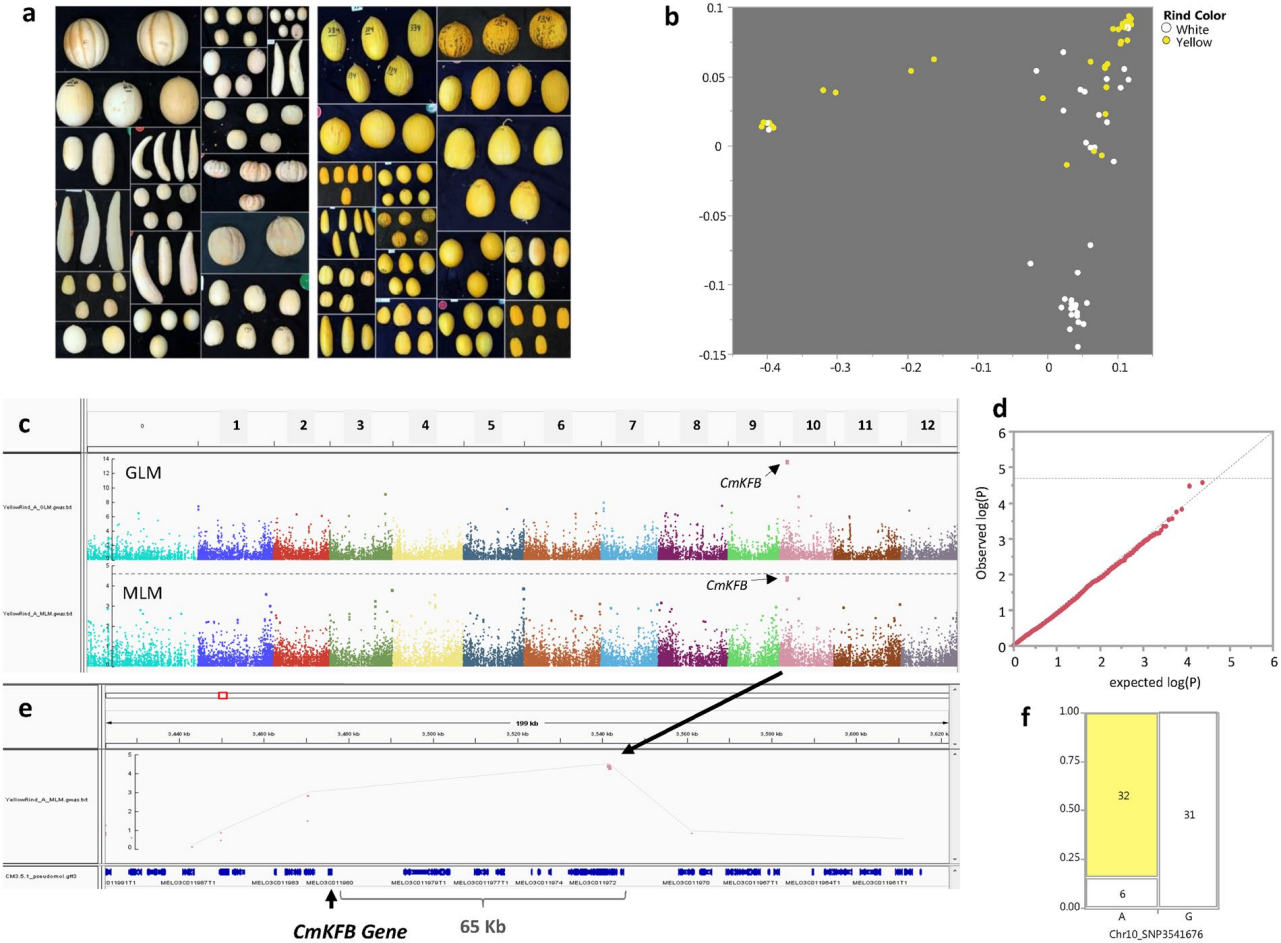
Mapping of yellow rind trait. (**a**) Examples of some fruits with yellow and white rinds used for the analysis of the trait. (**b**) Yellow or white rind colors projected on genetic PCA plot. (**c**) Manhattan plot of GWA of rind color (both simple model (GLM) and population structure corrected model (MLM_Q + K) are presented). (**d**) Quantile-quantile (Q-Q) plot for distribution of P values at the MLM_Q + K model. The negative logarithm of the observed (y axis) and the expected (x axis) P value is plotted for each SNP (dot). The gray dashed line indicates the null hypothesis. (**e**) Zoom in on chromosome 10 peak where causative gene *CmKFB* (MELO3C011980)^10^ is shown. (**f**) Contingency analysis for distribution of rind color across alleles at Chr10_SNP3541676.

Melon accessions with yellow rind are present in our GWAS panel. We previously reported that the yellow color is caused by the accumulation of naringenin chalcone, a yellow flavonoid pigment^37^. A Kelch domain-containing F-box protein coding gene (*CmKFB*) on chromosome 10 was identified as causative for the yellow rind phenotype in yellow casaba melon accession (*C. melo*, var *inodorus*)^10^. We characterized the collection for rind color through visual scoring and selected only smooth rind (without netting that could mask the rind color) accessions that are either yellow or white/cream (Fig. 6a) for association analysis. Sixty-nine accessions remained and were included in the yellow rind association analysis, 37 accessions with white/cream rind and 32 with yellow rind. Projection of this trait on the genetic PCA plot is showing population structure dependence for the phenotypic distribution with a high proportion of the white rind in the *reticulatus* group and most yellow accessions in *inodorous*. However, both *agrestis* and *melo* sub-species show polymorphism for this trait (Fig. 6b). GWAS was performed using both GLM and MLM approaches and the effect of population structure control on genome-wide significance levels are evident (Fig. 6c,d). While sample size used for this analysis is fairly small (due to the exclusion of netted and orange or green rind accessions), the strongest genome-wide effect that we found for this trait, on chromosome 10, was still marginally significant at FDR30% (P = 4 × 10^−5^, MLM). Zooming in on this region showed two SNPs at positions 3,541,676 and 3,541,866 (190 bp apart) that are in complete LD (R^2^ = 1). These SNPs, that showed the strongest association with yellow rind trait, are located 65 Kb and 8 genes away from the *CmKFB* gene^10^ (MELO3C011980: Chr10 3,475,253–3,476,386, Fig. 6e). SNP3541676 has MAF of 0.45 across the ‘rind color’ panel and it is associated with rind color in 90% of the accessions (Fig. 6f). To further validate SNP3541676 and SNP3541866 at the QTL peak, we genotyped the yellow and white rind accessions using PCR marker at the *CmKFB* gene^10^ and good co-segregation with the peak markers was shown (Figure S13). Local LD decline pattern for this genomic region fits with the genome-wide average and supports the obtained resolution (Figures S2 and S14). It is important to note that yellow rind phenotyping was based here only on visual observation and not supported by chemical analysis of flavonoids or carotenoids. Therefore, it could be that some of the fruits defined as yellow in fact accumulate carotenoids rather than naringenin chalcone. Moreover, melons also exhibit genetic variability for waxiness of the cuticle^38, 39^. A waxy epicuticular layer can mask rind colors and lead to modified visual phenotyping. These factors may have introduced some ‘noise’ to our mapping, further reducing the significance of the Chr10 QTL.

### Characterization and mapping of fruit shape

**Figure 7:**
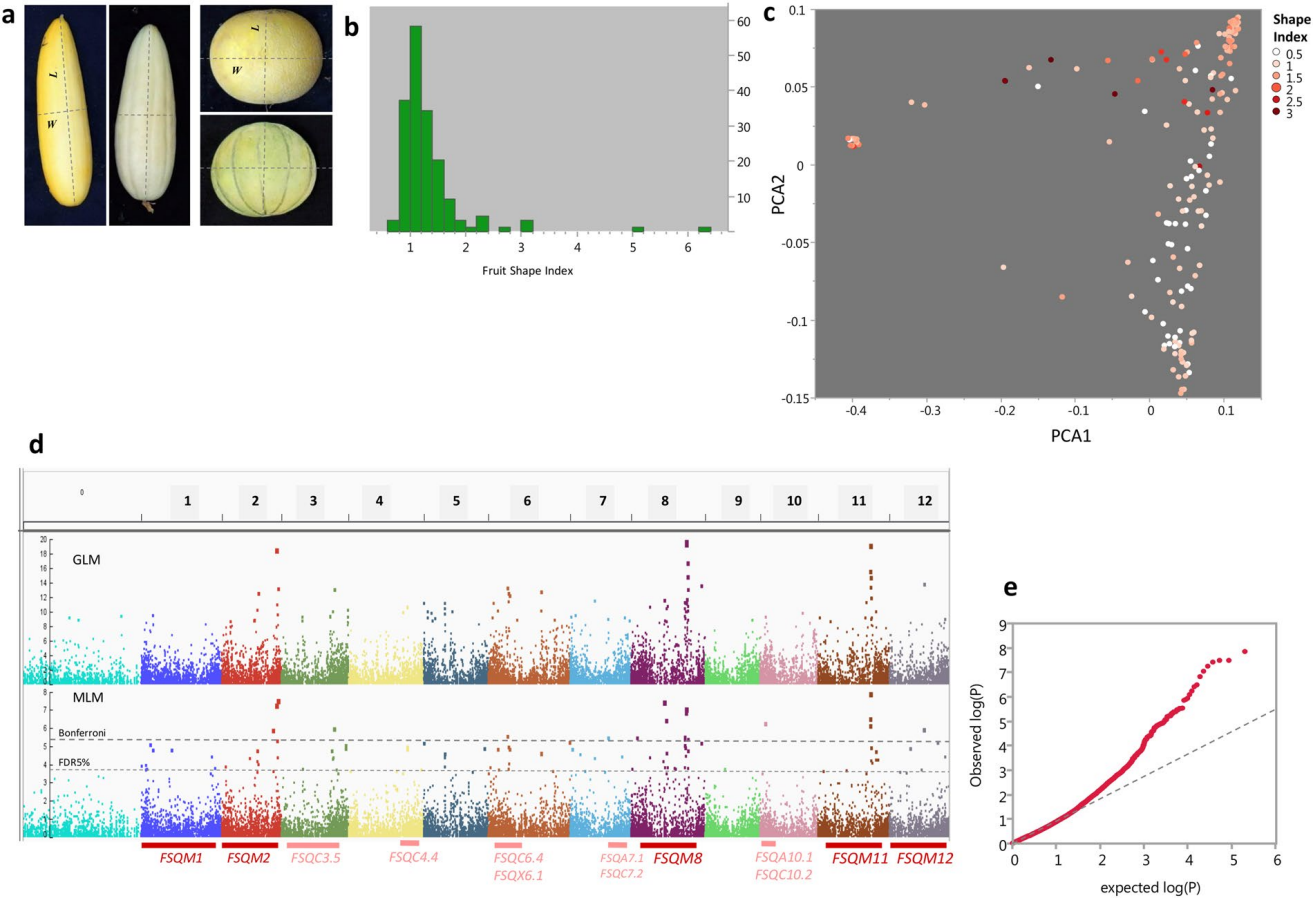
Characterization and mapping of fruit shape. (**a**) Examples of elongated and round fruits from the collection and the measurements taken for shape analysis. (**b**) Frequency distribution of fruit shape index for accession means. (**c**) Fruit shape index projected on genetic PCA plot. (**d**) Manhattan plots of GWA of fruit shape index. Upper panel: GLM analysis. Lower panel: MLM_Q + K analysis. Dashed gray horizontal lines are genome-wide significance thresholds adjusted for multiple comparisons using FDR5% and Bonferroni corrections. Horizontal bars bellow the plot indicate names and positions of previously mapped QTLs^5^. Dark red are meta-QTLs. Pink are QTLs identified in one or two experiments. (**e**) Quantile-quantile (Q-Q) plot for distribution of P values at the MLM_Q + K model. The negative logarithm of the observed (y axis) and the expected (x axis) P value is plotted for each SNP (dot). The gray dashed line indicates the null hypothesis.

Extensive diversity is present in *C. melo* for fruit size and shape. Fruit weight varies 100-fold within our collection (50 g–5000 g) and fruit shape index (ratio between length and width) varies between 0.66 and 6.3 (10-fold) across the panel (Fig. 7a,b, Table S2, Figure S15). Fruit shape is an important trait from breeding perspective as, alongside other fruit attributes such as rind netting, flesh color and ripening behavior, it defines horticultural and marketing groups. While the components of fruit shape (fruit length and width) may be influenced by environmental conditions (as they reflect growth rate), shape index seem to be a very coordinated and conserved attribute. Furthermore, fruit shape is programmed early on in fruit development process as there is good correlation between ovary and mature fruit shape indexes in melon^40, 41^. The collection was characterized for fruit shape through image-analysis-based measurements taken on digital scans of longitudinal fruit sections (Fig. 7a, see materials and methods). Fifteen fruits per accession were measured and the average shape index was calculated. The extensive diversity and wide sampling resulted in very high heritability obtained for this trait (H^2^ = 0.95, Figure S15). Projection of fruit shape means on the genetic PCA revealed the distribution of shape variation across the genetic landscape and the effect of population structure on phenotypic distribution (Fig. 7c). Most of the elongated fruits (index > 1.5) belong to *C*. *melo* ssp. *melo* var. *inodorous* and *flexuosus*, and *C*. *melo* ssp. *agrestis* var. *makua*. Next, we mapped fruit shape through GWA analysis. Significant SNPs in MLM analysis (at FDR5%) were found on all chromosomes (Fig. 7d, Table S3). We then compared our significant SNPs with previously mapped fruit shape QTLs from multiple studies that have been summarized into a consensus map^5, 12^. The polygenic architecture of fruit shape observed here is in agreement with these previous results where fruit shape QTLs were mapped in different studies to eleven out of the twelve melon chromosomes. Monforte *et al*.^5^ identified meta-QTLs for fruit shape on five chromosomes where common intervals were identified in several studies. We found significant SNPs (at Bonferroni adjusted threshold) on four of these chromosomes (FSQM-2, 8, 11 and 12, Fig. 7d,e and Table S3). On chromosomes 3, 6, and 10 we found significant SNPs that overlapped with QTLs that were reported in a previous study (FSQC3.5, FSQC6.4, FSQX6.1, FSQA10.1, FSQC10.2^5^). Interestingly, large effects appear in higher frequencies for SNPs with MAF < 0.1 (Figure S16), while the distribution of allele frequencies is relatively uniform across our genotyping set filtered to MAF > 0.05 (Figure S17). Similar patterns of negative correlations between polymorphism effect size and allele frequency were also shown for multiple traits in maize^42, 43^.

## Discussion

The power of combining NGS-based genotyping and GWAS for achieving mapping to the gene level resolution was demonstrated in several recent studies in plants^44, 45^. The ability to simultaneously screen diverse germplasm collections and map traits through GWAS was described as a mean to improve tomato flavor^46^, or identify candidate-genes for manganese efficiency in barely^47^.

The current study is aimed at establishing melon diversity collection as a permanent germplasm resource for discovery of phenotypic variation and genetic mapping of traits. We performed phenotypic and genotypic analyses to test and demonstrate the genetic resolution and overall effectiveness of this platform. The panel used in this study is composed of 177 accessions that represent large portion of the genetic and phenotypic diversity within *C. melo*. The collection was genotyped with 23,931 SNPs that cover all 12 melon chromosomes at an average density of ~1 SNP per 18 Kb. This density and coverage allowed us to calculate the extent of LD and show that LD decays on average across ~100 KB in this panel (Fig. 3), defining the potential mapping resolution and estimated number of markers required for GWAS in this crop. Since LD decay pattern reflects an average genetic attribute that describes theoretical mapping resolution, we performed real mapping of traits to test the actual resolution obtained. Three traits with high heritability, simple genetic basis and known causative genes were used as test cases: Sex-expression trait was mapped to chromosome 2, one gene away from the previously identified causative *CmACS-7* gene^35^. Major flesh color locus was mapped within the causative *CmOr* gene that was mapped earlier using bi-parental linkage analyses^7, 34^ and later cloned using candidate gene approach^9^. The yellow rind trait locus was mapped 65 KB from the causative *CmKFB* gene that was previously identified through RNA-Seq bulk-segregant analysis (BSA) on F3s^10^.

Thus, three independent single gene traits were successfully mapped in the current study to narrow genomic intervals, where the most significant SNPs identified for each trait were located within less than 100 Kb windows from the known, previously mapped, causative genes. LD around these mapped trait loci decayed within the expected physical distance calculated at the whole genome level and allowed us to define confidence intervals containing less than 20 annotated open-reading frames (ORFs) in these three examples.

Fruit shape is highly polymorphic trait that has been extensively studied in melon and shown to be governed by multiple QTLs^2–5^. We analyzed fruit shape variation across the diversity panel and mapped QTLs for this trait. The extended variation and high heritability enabled the identification of multiple significant SNPs across all chromosomes (Fig. 7d, Table S3). Strong signals were detected in chromosomes 2, 8 and 11 where meta-QTLs were previously described^5^, as well as significant effects in other genomic regions mapped in specific studies. Availability of a reference genome for melon^32^ facilitates effective comparative mapping through alignment of results from different studies into a common framework^48^. The added value of using multi-allelic population is demonstrated here for fruit shape through the simultaneous identification of QTLs that were previously found in different populations.

Visual projection of phenotypic variation on the genetic landscape (expressed as 2D PCA plots) is shown here as a useful instrument to evaluate the relation between population structure and trait variation and to gain perspective about the history of allelic variation in target traits. For example, one can hypothesize that the *CmOr* gene dominant mutation, which governs the accumulation of carotenoids (mainly β-carotene) and orange flesh color^9^, most likely occurred during sub-speciation as the orange allele is present only in one accession of the *ssp. agrestis* group in our collection (6%) while the frequency of the Orange allele at the *ssp. melo* group is 41%. The genotypic data localize the light-orange-flesh *ssp. agrestis* accession (PI414723) intermediate between the two sub-species, supporting this hypothesis (Fig. 5c). A second flesh color variant, *wf*, that is associated with the transition from white to green flesh and was previously described and mapped to chromosome 8^3, 49^ is evident only within the *ssp. melo* group. The effect of breeding selection is reflected by the clear division between color categories within *C. melo ssp. melo* where most orange accessions belong to the *reticulatus* and *cantalupensis* groups and most white accessions are among *inodorous*. This trait is now readily transferred across genetic backgrounds using marker-assisted selection and it is expected that distribution of flesh color across horticultural groups will be more uniform with the development and distribution of new product combinations.

Population structure and cryptic relatedness may lead to false-positive discoveries in association analyses^50^. The correlation between population structure and trait distribution is defining the expected rate of spurious associations and the impact of correcting for structure and relatedness on distribution of P values^21^. We show in the current study the varying effect of population structure on GWA results across different traits in melon. The most striking example is ovary hairiness, spreading versus appressed, which determined subdivision of *C. melo* into two subspecies, ssp. *melo* (spreading) and ssp. *agrestis* (appressed)^51^. As expected, hairiness phenotypic distribution is completely correlated with sub-specific division and therefore excess of significant effects (false positive associations) are found genome-wide (Figures S18). Another trait where distribution of phenotypes is confounded with population structure is flesh color (Fig. 5c). The prominent difference in distribution of GWA P values between GLM and MLM analyses in both of these traits (Fig. 5d,e, Figure S18D,E, Figure S19) is a clear evidence for that. While the control for population structure reduced the false-discovery ‘noise’ but did not exclude the detection and mapping accuracy for the major flesh color gene *CmOr*, it has most likely eliminated other true-positive associations that explain quantitative variation in color^6, 7, 11^. Flesh color and ovary hairiness are therefore examples for traits where the confounding effect of population structure reduce the power of association mapping and highlight the advantage of non-structured linkage designs. Similar situation was described for flowering time and other traits in maize^52^. This limitation of association mapping approach has led to the development and implementation of alternative multi-allelic designs in plant^53^ and animal^54^ genetics that combine association and linkage properties. In maize, Nested-Association Mapping (NAM) and Multi-parent Advanced Generation InterCrosses (MAGIC) designs were demonstrated as efficient platforms for high resolution trait dissection^55, 56^. MAGIC design was also recently used in tomato to detect candidate SNPs underlying QTLs^57^.

**Figure 8:**
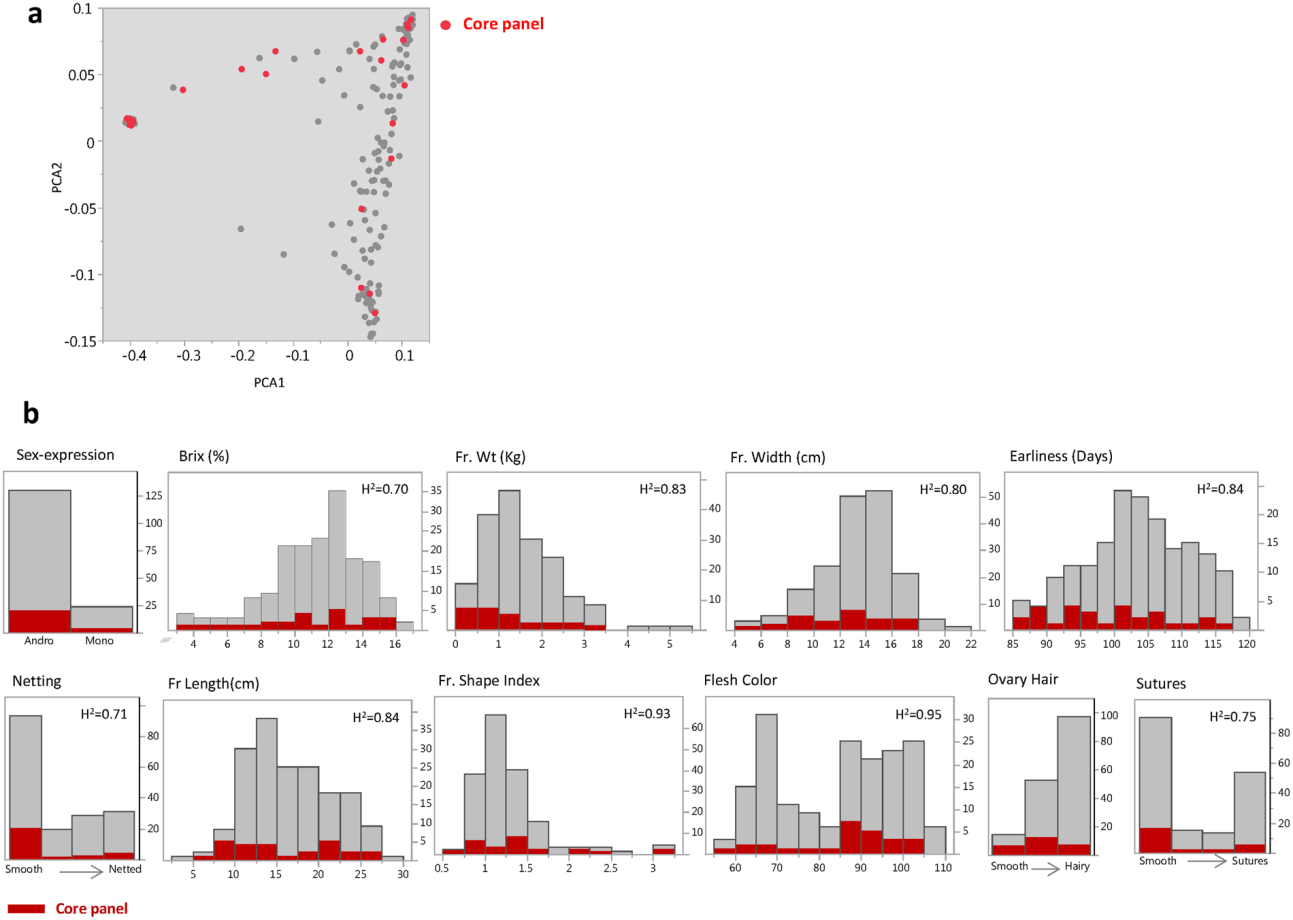
Core-panel (25 accessions) that represent genotypic and phenotypic spectrums across the diverse collection. (**a**) Two-dimensional genetic PCA plot. Core-panel accessions are shown in red. (**b**) Frequency distributions of various phenotypic traits. Core-panel accessions are highlighted in red.

Advances in genotyping technologies, computational and statistical tools are supporting the implementation of multi-allelic designs. The ability to simultaneously capture a wide spectrum of allelic variation for genetic mapping is appealing compared to traditional bi-parental designs, particularly in cases where multiple traits are targeted in parallel. The goal of the current study was to evaluate the potential of using diverse collection for LD mapping in *Cucumis melo*. The results presented here provide demonstration for the effectiveness of the melon diversity collection and GWAS approach to map simple traits to candidate-gene level. Based on experience and lessons from other crops, the next logical step to promote the efficient dissection of complex traits in melon would be the development of multi-allelic segregating populations that will overcome the inherent limitations of GWAS and can serve as a community resource for broader comparative genetics within the Cucurbitaceae. The genotypic and phenotypic infrastructure laid in this study can support the selection of balanced representative core panel for that purpose as shown for our current 25 founders core panel (Fig. 8, materials and methods), where for both phenotypic and genotypic plots, selected accessions are distributed uniformly and capture the diversity spectrum.

## Materials and Methods

### Plant materials and field experiment

The Newe-Ya’ar melon diversity collection used in this study is comprised of 177 inbred accessions derived from many countries and encompassing the two melon subspecies (ssp. *agrestis* and ssp. *melo*) and 11 horticultural groups (Fig. 1, Table S1, Figures S3 and S4). Seeds of each of the accessions were sown in seedling trays in early March, 2015. Seedlings were transplanted in early April to the field at Newe Ya’ar (32°43′05.4″N 35°10′47.7″E). The seedlings were spaced 50 cm apart, on raised beds covered with silver-colored plastic mulch (Ginnegar), 200 cm between bed centers. Soil type was grumusol, and the plants were drip-irrigated and drip-fertilized, to approximately 180 L/m^2^ over the course of the growing season. Each accession was represented by three plots of five plants each; in a randomized block design (RCBD).

The bi-parental populations that were used as reference for phenotypic variation spectrum in this study are created with lines that are part of the diverse collection and are shown in Figure S1. The three populations are: (1) SAS × DOYA population (120 F4 lines); cross between a sweet round-fruited accession (*C. melo ssp. agrestis*, var *makuwa*) and the non-sweet elongated pickling melon (*C. melo ssp. melo*, var *flexuosus*). (2) TAD × DUL population (166 RILs; F7); cross between an honey-dew line (*C. melo ssp. melo*, var *inodorus)* and an American cantaloupe melon (*C*. *melo ssp. melo*, var *reticulatus*). (3) PI414723 x DUL population^11^ (99 RILs); a wide cross between a *C. melo ssp. agrestis*, var *momordica* accession and an American cantaloupe melon (*C. melo ssp. melo*, var *reticulatus)*. These populations were grown during several years in the same location (Newe-Yaar) and similar growing regime.

Core panel of 25 founder lines for future multi-allelic segregating populations was selected based on multiple criteria derived from this study: initial tentative set (n = 40) was constructed to represent all the different horticultural groups in the diverse collection (based on traditional classification). Phenotypic profiles were then used as the second primary factor; the preliminary core set was projected on the distribution of the different traits to ensure phenotypic spectrum is well captured in the core panel (as illustrated in Fig. 8). Following required adjustments and narrowing of the set to n = 30, based on the first two steps, final set was selected to meet the 25 accessions target, taking into account maximum polymorphism information content (PIC) value and uniform distribution on genetic diversity plots (PCA and phylogenetic tree).

### Phenotyping

The collection was subjected to phenotypic characterization of fruits throughout the growing season to reflect variation in fruit development, with most effort on mature fruits. Phenotyping during fruit development was largely based on images taken at the field using digital camera. Representative female flowers and fruits from each accession were pictured once a week from anthesis till harvest. Images were tagged and used for manual annotations of fruit traits. Melons were harvested when fully ripe, as defined by number of days after anthesis, rind color and firmness in the non-climacteric accessions and by abscission of the fruit from the peduncle (fruit stem) in climacteric accessions. Five mature fruits per plot were sampled in multiple harvests and total of 15 fruits per accession were analyzed. Due to large variation in maturity time across the diverse collection, the field was walked through on a two-daily basis from middle of June till end of July for selective harvest of mature fruits. Melons were weighed and cut longitudinally for external and internal imaging. Internal side of all fruits was scanned using a standard document scanner (Canon, Lide120). Three fruits per plot were used for flesh color measurement (three reads per fruit) using hand colorimeter (Minolta Sensing Inc, Minolta Chroma Meter Model CR-400, Osaka, Japan). Flesh and rind samples were taken as bulks from at least three fruits per plot and immediately frozen in 50 ml tubes in liquid nitrogen, for further biochemical and molecular analyses. Fruit internal scanned images were analyzed using the Tomato-Analyzer software^36^ for the extraction of color, shape and size attributes.

### SNP genotyping

#### DNA isolation for GBS

Total genomic DNA extractions were performed on the177 accessions. DNA isolation was performed using the GenEluteTM Plant Genomic DNA miniprep kit (Sigma, St. Louis, MO). The quality of the DNA was analyzed by ND-1000 Spectrophotometer (Nanodrop Technologies, Wilmington, DE) and by electrophoresis on agarose gel. The concentration of DNA was estimated using Qubit® 2.0 Fluorometer (Life Technologies, Singapore) and Qubit® dsDNA BR Assay Kit (Life Technologies, Eugene, OR).

#### GBS analysis

DNA was shipped to the Institute for Genomic Diversity facility at Cornell University for GBS. GBS 96-plex libraries were prepared using the restriction enzyme ApeKI, following an established protocol^31^. Fragments were sequenced on an Illumina HiSeq. 2500 as 100 bp, single-end reads and aligned to the reference genome of *C. melo* ^32^ available at https://melonomics.net/files/Genome/Melon_genome_v3.5.1/. TASSEL pipeline v3.0.173 was used for sequence alignment and single nucleotide polymorphism (SNP) calling^58^. Further filtration was performed using TASSEL v5.2.33^59^; SNP list was filtered to MAF > 0.05 and maximum of 6% missing data per site.

#### Validation of trait peak markers

Association results for flower sex-expression, flesh color and yellow rind were validated by genotyping polymorphisms at the causative genes: polymorphism at the *CmACS-7* gene (MELO3C015444) was genotyped using CAPs marker adapted from Boualem *et al*.^35^. A 234 bp amplicon was produced (Forward primer: AGATTCGCCGTATTTTGCTG, Reverse primer: CCCTCACAATTTTCCTCCAA), cleaved with *Alu I* restriction enzyme and seperated on a 2% agarose gel. For *CmOr* gene (MELO3C05449) polymorphism was genotyped based on the SNP described by Tzuri *et al*.^9^. 500 bp amplicons (Forward: CTCCTTGGTTTTCTTCATG, Reverse: CAACAAAACCCATCAAGTC) were sequenced and aligned across all samples. SNP at position 160 was called. Polymorphism at the *CmKFB* gene was genotyped using a protocol adapted from Feder *et al*.^10^. A 81 bp product was amplified (Forward: AACACTCAAAATTCACTAAATGGTCT, Reverse: TGTCGTAATTTTAATTTTACTTATTTTTATC) and a 23 bp indel differentiating between the alleles was scored on a 2.5% agarose gel.

### Data analysis

#### Population structure, Kinship and LD analysis

TASSEL software (V 5.2.33)^59^ was used to estimate the relatedness matrix of pairwise kinship (k matrix) from the filtered SNP dataset using the Centered_IBS method^60^. Intra-chromosomal LD between pairs of sites was calculated in TASSEL on SNP set filtered to MAF > 0.15 to ensure reliable estimates. Phylogenetic tree was built using the neighbor-joining function in MEGA^61^. An admixture-based clustering model implemented in the software STRUCTURE^33^ was used to infer the genetic structure of the collection. Ten independent runs for each K value ranging from 1 to 10 were performed with a burn-in length of 100,000 and 100,000 Markov chain Monte Carlo (MCMC) repeats after burn-in. The optimal subpopulations number was calculated from the second order rate of change of likelihood (delta K method)^62^.

#### GWA mapping

In this study, four models (run in TASSEL) were used for the association analysis: the first used a generalized linear model (GLM) without any consideration for population structure; the second was GLM + Q where inferred ancestry of individuals (Q matrix) is used as covariate in the model; the third model was mixed linear model (MLM) using kinship matrix (*k*; random effect based on the genetic relatedness across all accessions) and the fourth model was MLM using both population structure (Q matrix) and relatedness (kinshp matrix).This model considered population structure and cryptic relationships thereby minimizing false positives and increasing the statistical power. Supplementary Figure S19 is showing Quantile-quantile (Q-Q) plots where distributions of P values at the different models are compared to the expected null hypothesis distribution. Significance threshold was corrected for multiple comparisons using the FDR approach^63^. For fruit shape GWA analysis, stringent Bonferroni correction was used to control for multiple comparisons.

## Electronic supplementary material

Supplumentarry Information

Supplementary_Table S1

Supplementary_Table S2

Supplementary_Table S3

Supplementary_Table S4
