# Supplementary material for: Genome-Wide Linkage-Disequilibrium Mapping to the Candidate Gene Level in Melon (Cucumis melo)
Source: Sci Rep. 2017 Aug 29;7:9770. doi: 10.1038/s41598-017-09987-4 (PMC5575340; doi:10.1038/s41598-017-09987-4)
Supplement: Supplementary file 1 — Supplumentarry Information [file 41598_2017_9987_MOESM1_ESM.pdf]

Supplementary information

Genome-Wide Linkage-Disequilibrium Mapping to the Candidate Gene Level in Melon (*Cucumis melo)*

Amit Gur[1*], Galil Tzuri[1], Ayala Meir[1], Uzi Sa'ar[1], Vitaly Portnoy[1], Nurit Katzir[1], Arthur A. Schaffer[2], Li Li[3], Joseph Burger[1] and Yaakov Tadmor[1]

[1]Plant Science Institute, Agricultural Research Organization, Newe Ya'ar Research Center, P.O. Box 1021, Ramat Yishay 3009500, Israel. [2]Plant Science Institute, Agricultural Research Organization, The Volcani Center, P.O. Box 15159, Rishon LeZiyyon 7507101, Israel. [3]Robert W Holley Center for Agriculture and Health, USDA-ARS, Plant Breeding and Genetics Section, School of Integrative Plant Science, Cornell University, Ithaca, New York 14853, USA.

*Correspondence should be addressed to A.G. (e-mail: amitgur@volcani.agri.gov.il)

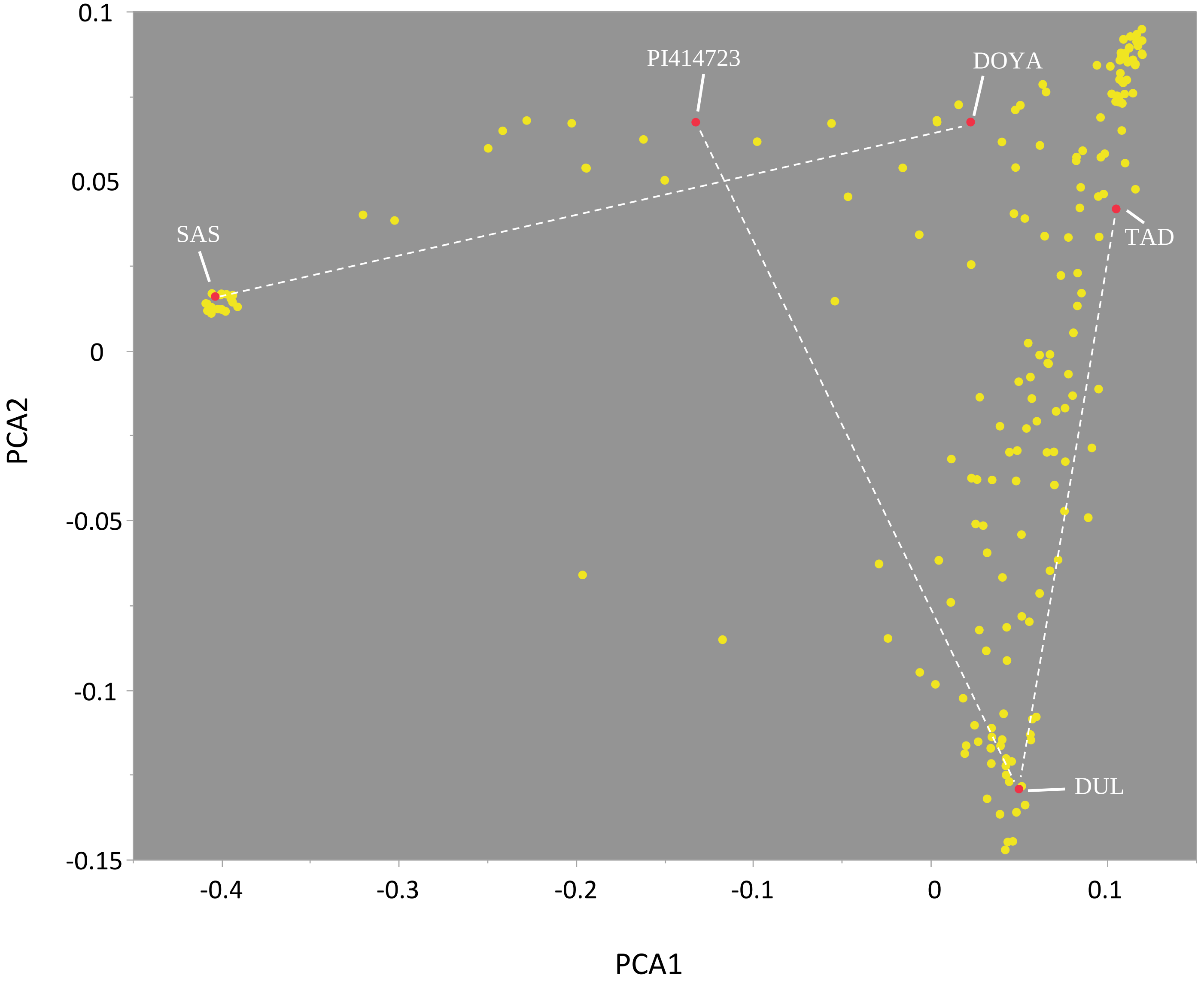

Supplementary Figure S1: **Parental lines of the 3 bi-parental populations highlighted on the genetic PCA analysis**. Crosses are shown in dashed white lines. SAS: C. *melo* ssp. *agrestis*, var *makuwa* . PI414723: C. *melo* ssp. *agrestis*, var *momordica*. DOYA: C. *melo* ssp. *melo*, var *flexuosus*. TADA*: C. melo ssp. melo, var inodorus*. DUL: C. *melo* ssp. *melo*, var *reticulatus*

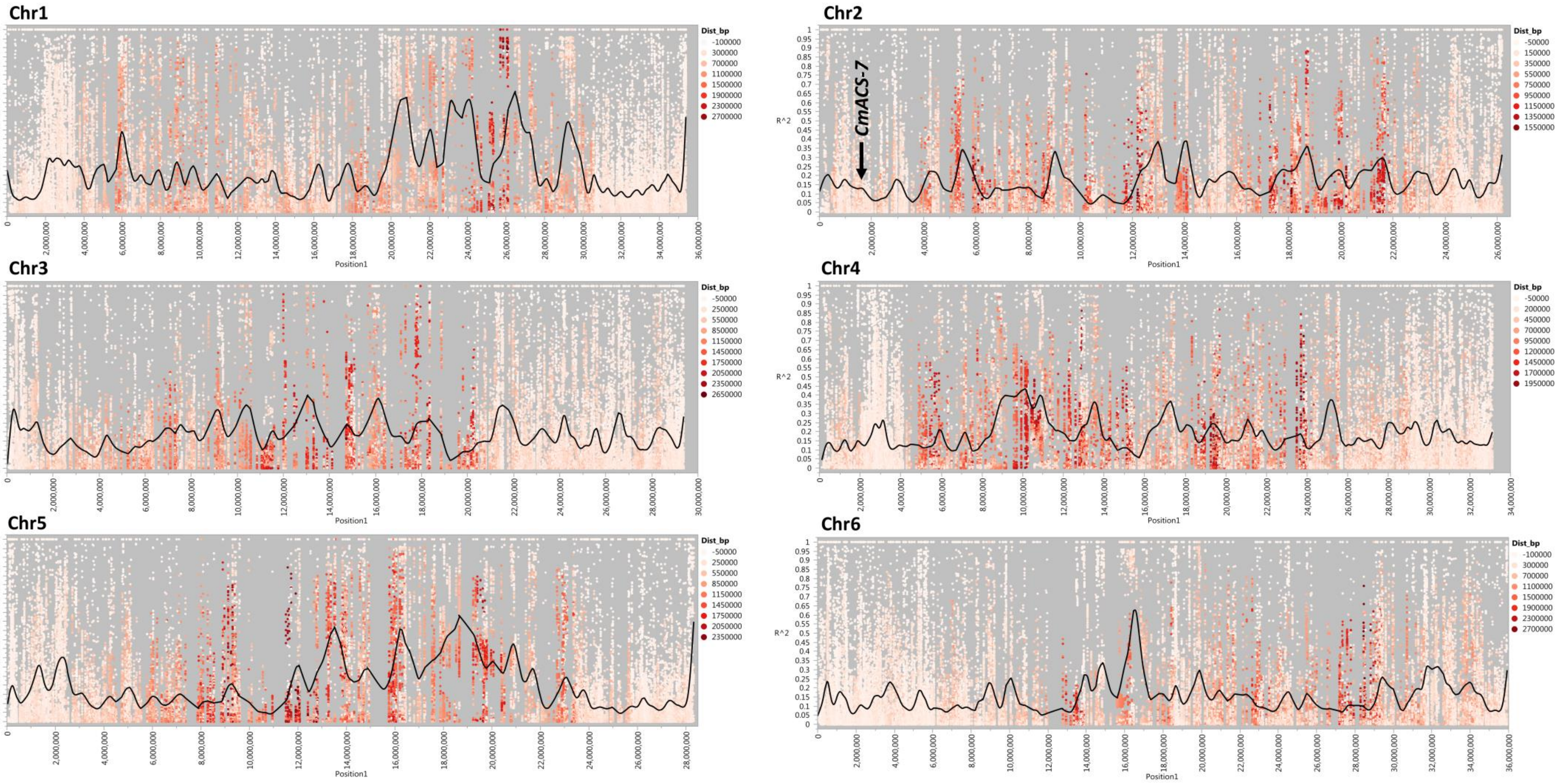

Supplementary Figure S2: **LD (R^2) by physical position across 12 melon chromosomes.**
LD between intra-chromosomal SNP pairs is plotted (Y axis) by the physical genomic position of first SNP in each pair. Distance between SNPs in each pair is indicated using white-to-red color scale.

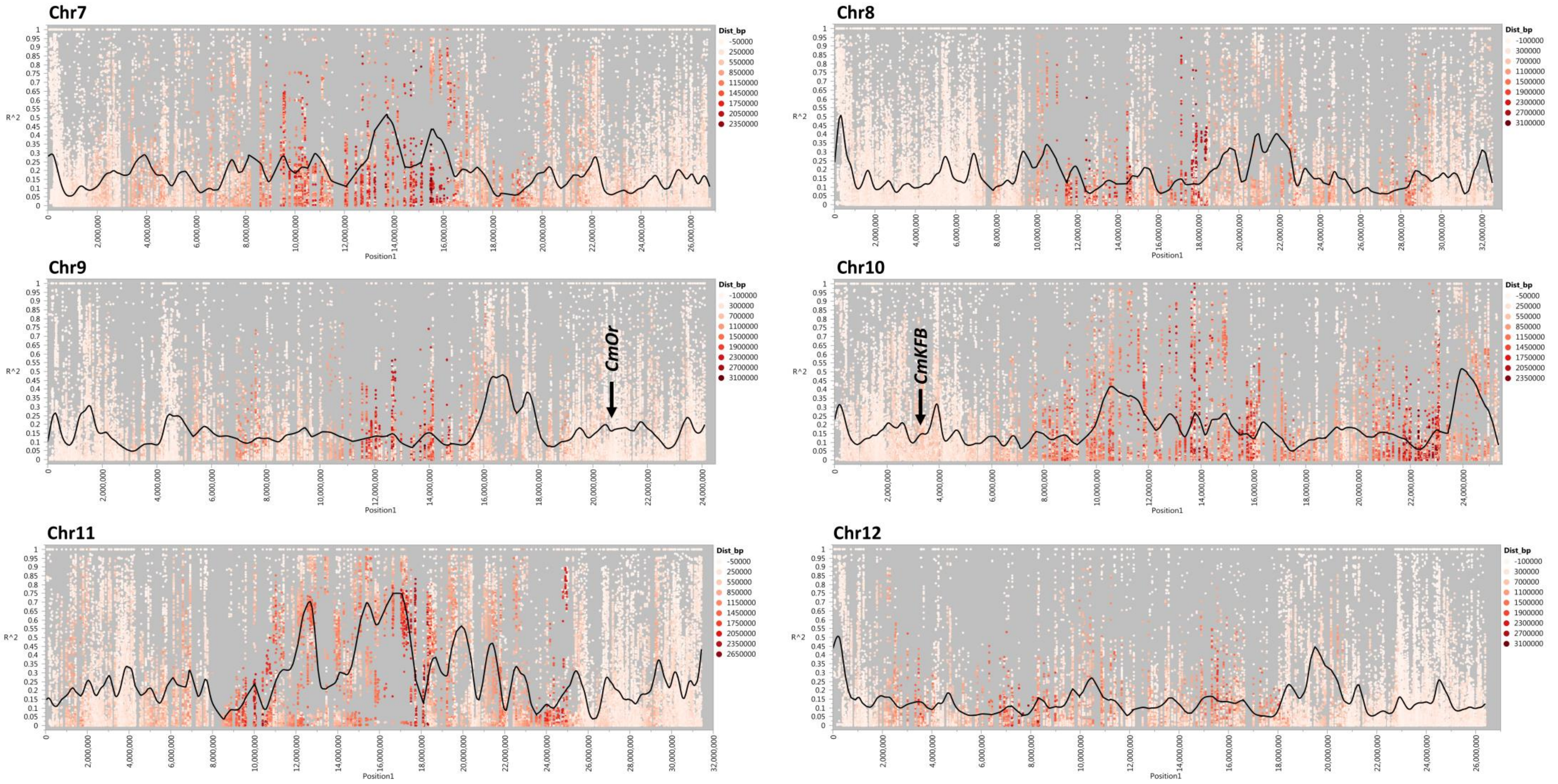

Supplementary Figure S2: **LD (R^2) by physical position across 12 melon chromosomes.**
LD between intra-chromosomal SNP pairs is plotted (Y axis) by the physical genomic position of first SNP in each pair. Distance between SNPs in each pair is indicated using white-to-red color scale.

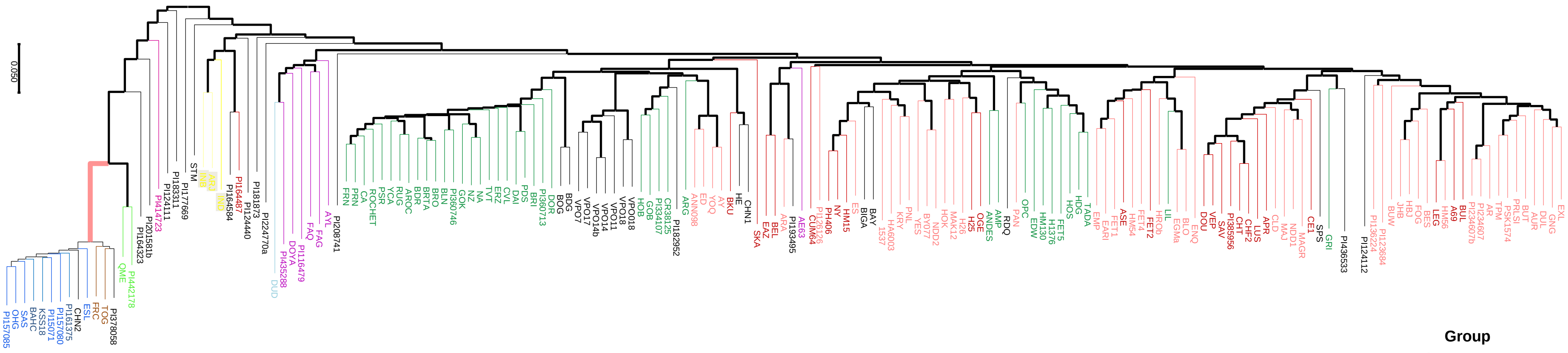

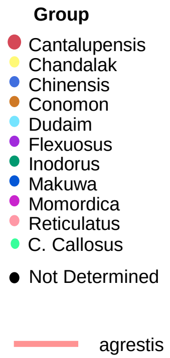

Supplementary Figure S3: **Neighbor-joining phylogenetic tree of 177 accessions.**
Tree was built based on distance matrix calculated using 23,931 SNPs.

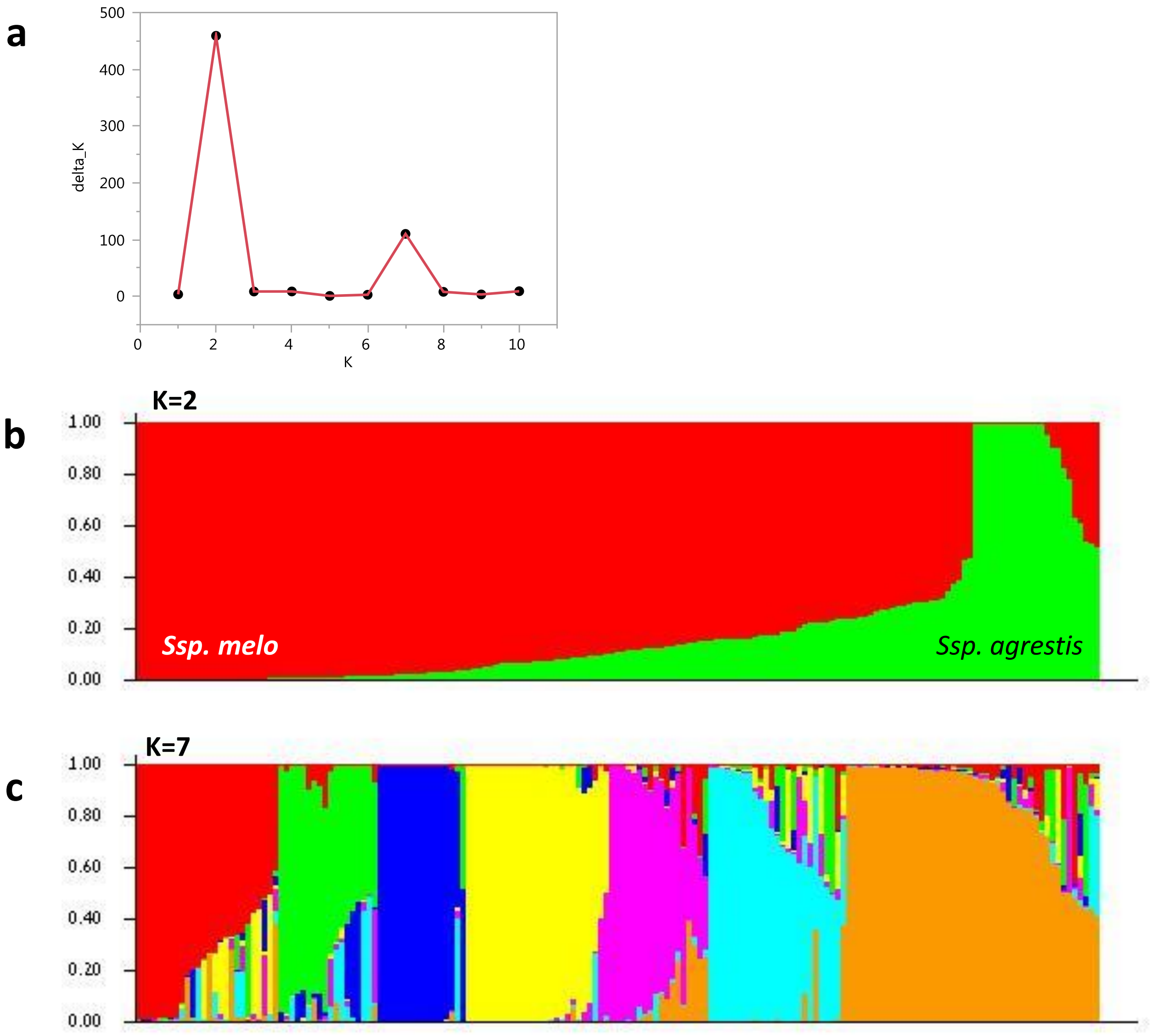

Supplementary Figure S4: **Population structure analysis.** Results are shown for the minimum number of subpopulations (K) which sufficiently define genetic variation. Each individual is represented by a vertical line. (**a**) detection of optimal subpopulations using the delat K method (Reference 62). Plot of delta K for K = 1–10. (**b**) Population structure for K=2, in consensus with the ssp. *melo* and ssp *agrestis* division. (**c**) Population structure obtained at K=7.

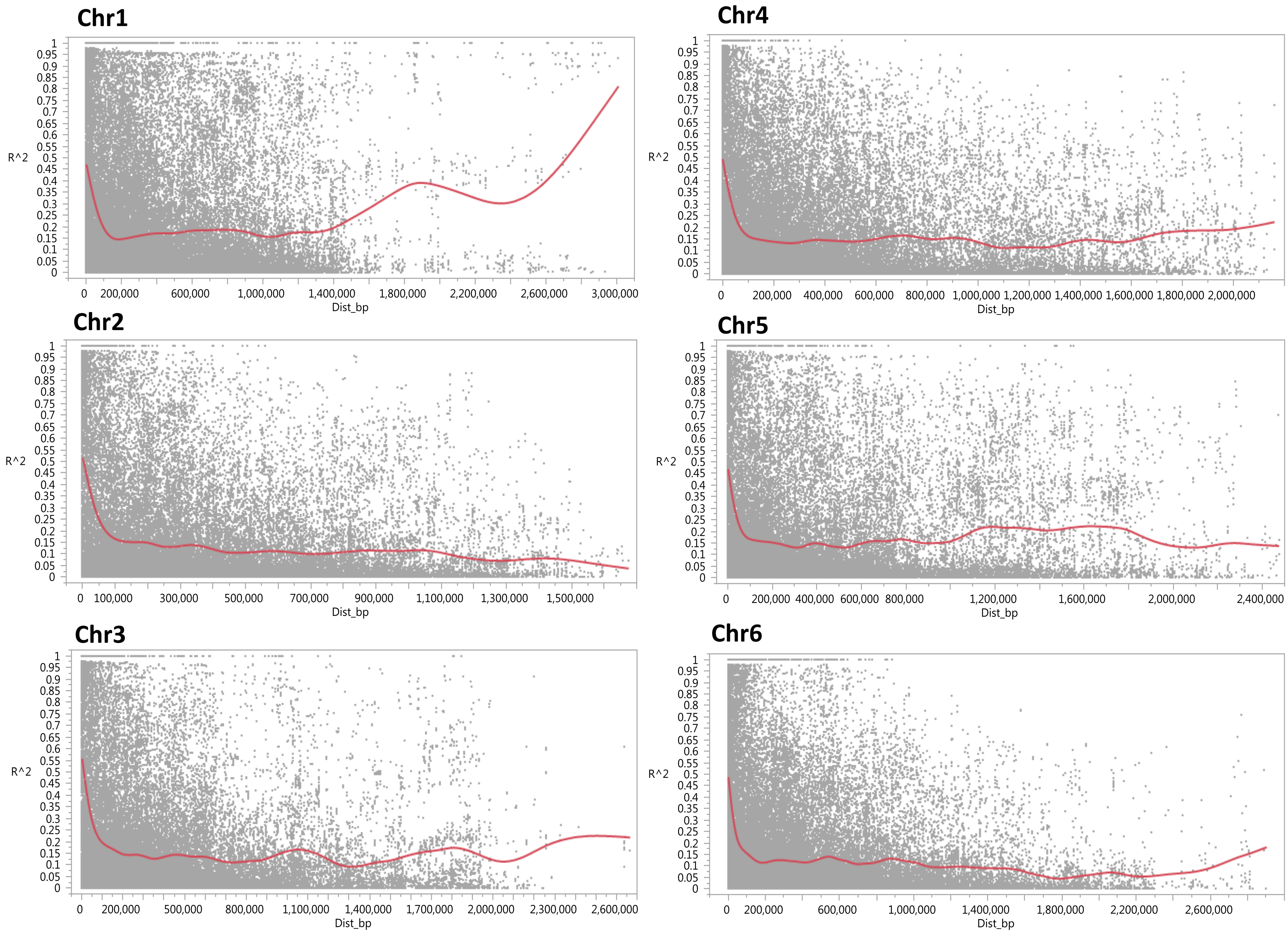

Supplementary Figure S5a: **LD decay plots by chromosomes across 177 accessions**. R^2 between intra-chromosomal marker pairs plotted against the physical distance between them.

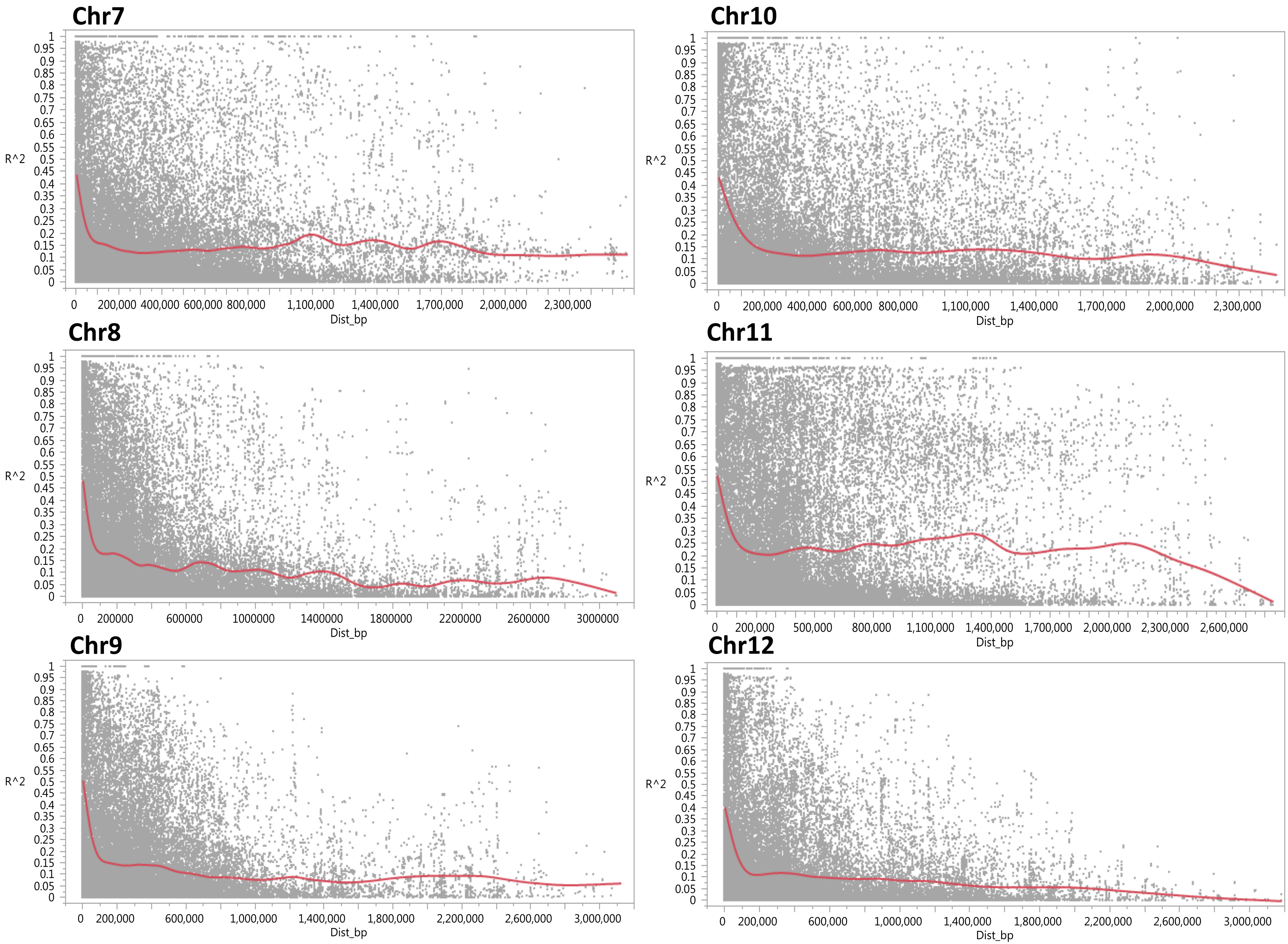

Supplementary Figure S5a: **LD decay plots by chromosomes across 177 accessions**. R^2 between intra-chromosomal marker pairs plotted against the physical distance between them.

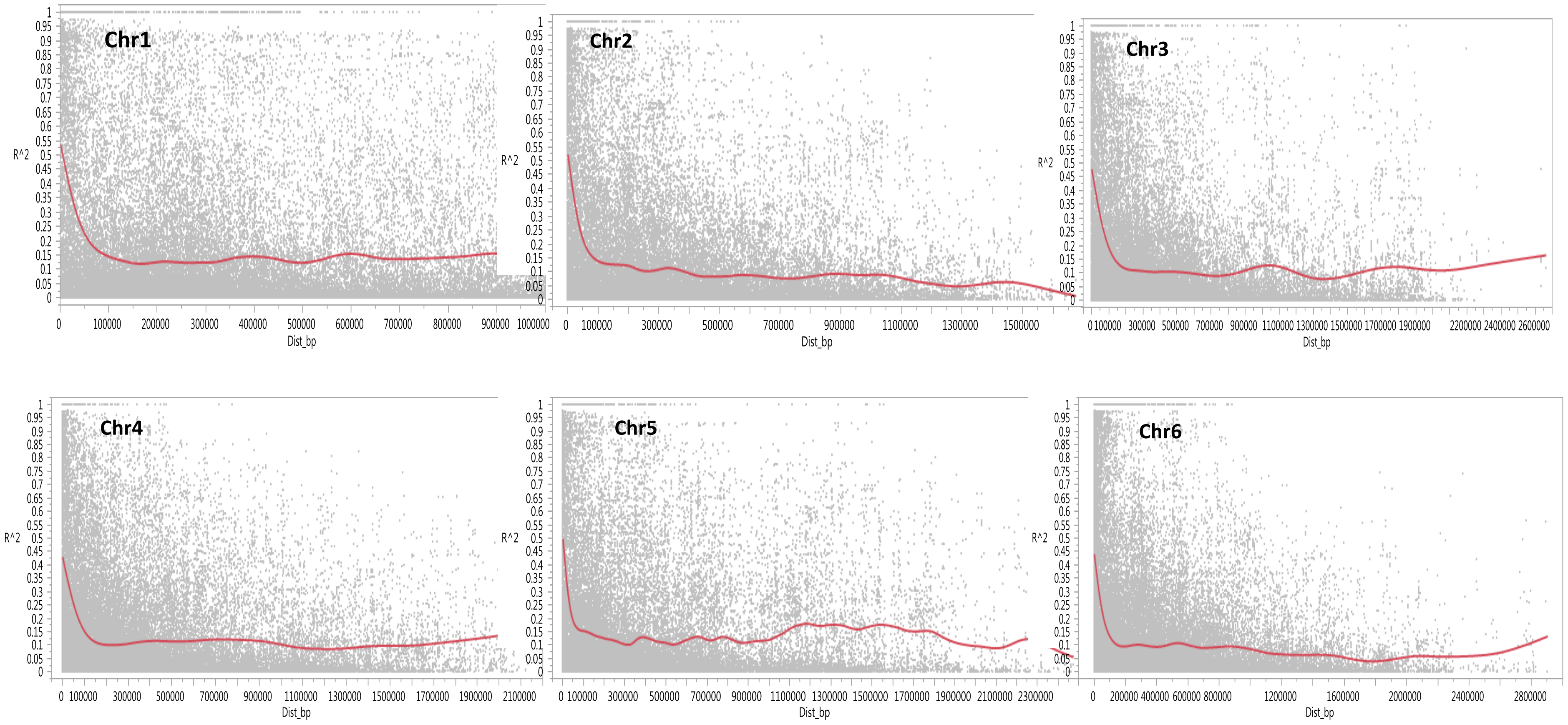

Supplementary Figure S5b: **LD decay plots by chromosomes within the *ssp. melo* group**. R^2 between intra-chromosomal marker pairs plotted against the physical distance between them.

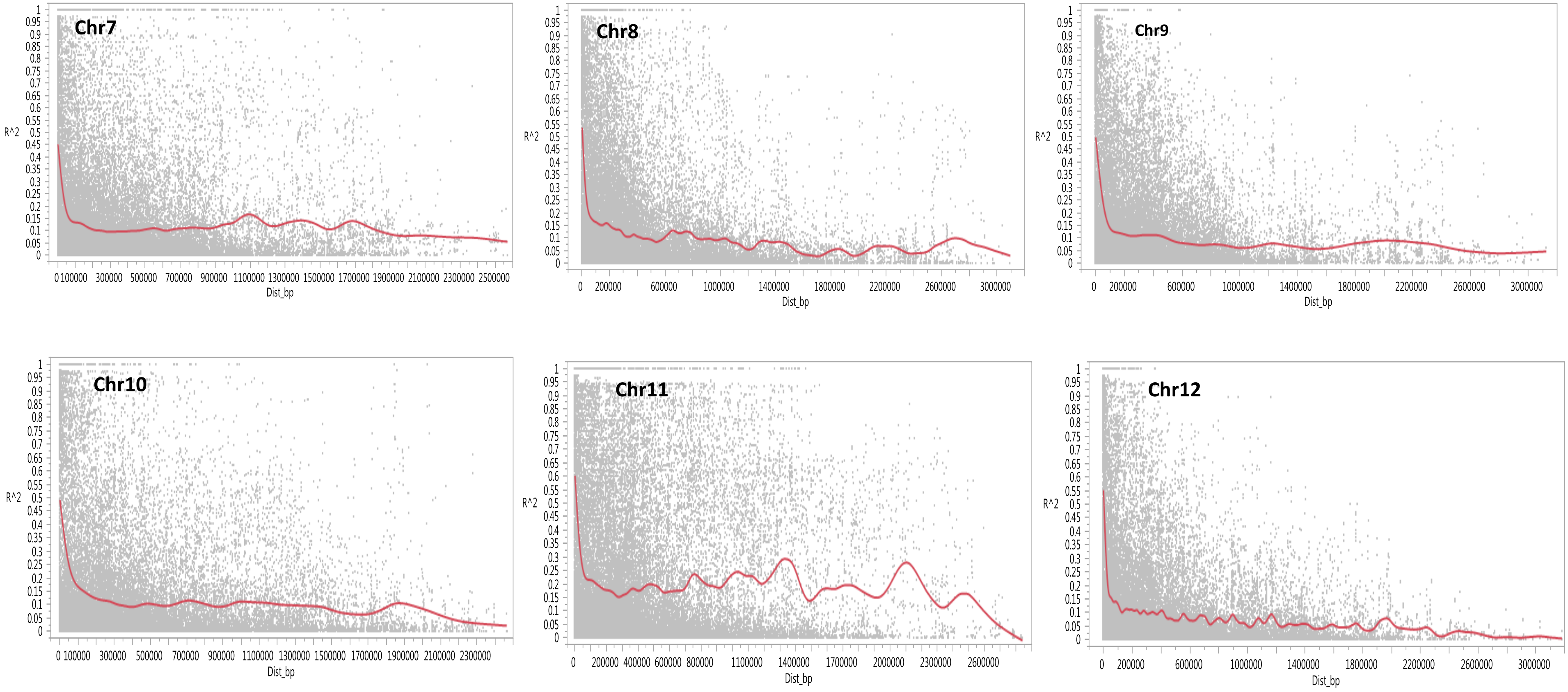

Supplementary Figure S5b: **LD decay plots by chromosomes within the *ssp. melo* group.** R^2 between intra-chromosomal marker pairs plotted against the physical distance between them.

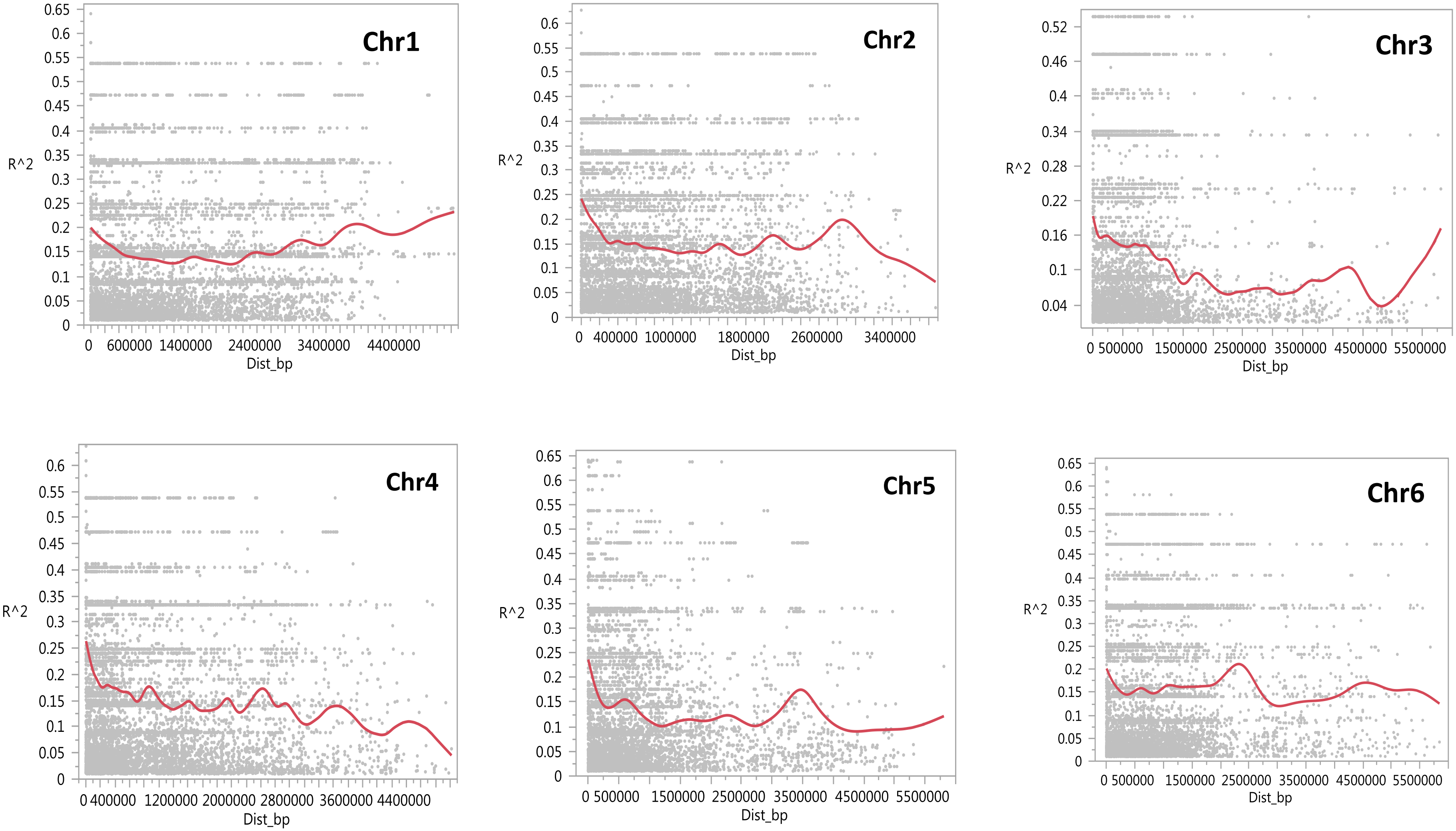

Supplementary Figure S5c: **LD decay plots by chromosomes within the *ssp. agrestis* group**. R^2 between intra-chromosomal marker pairs plotted against the physical distance between them.

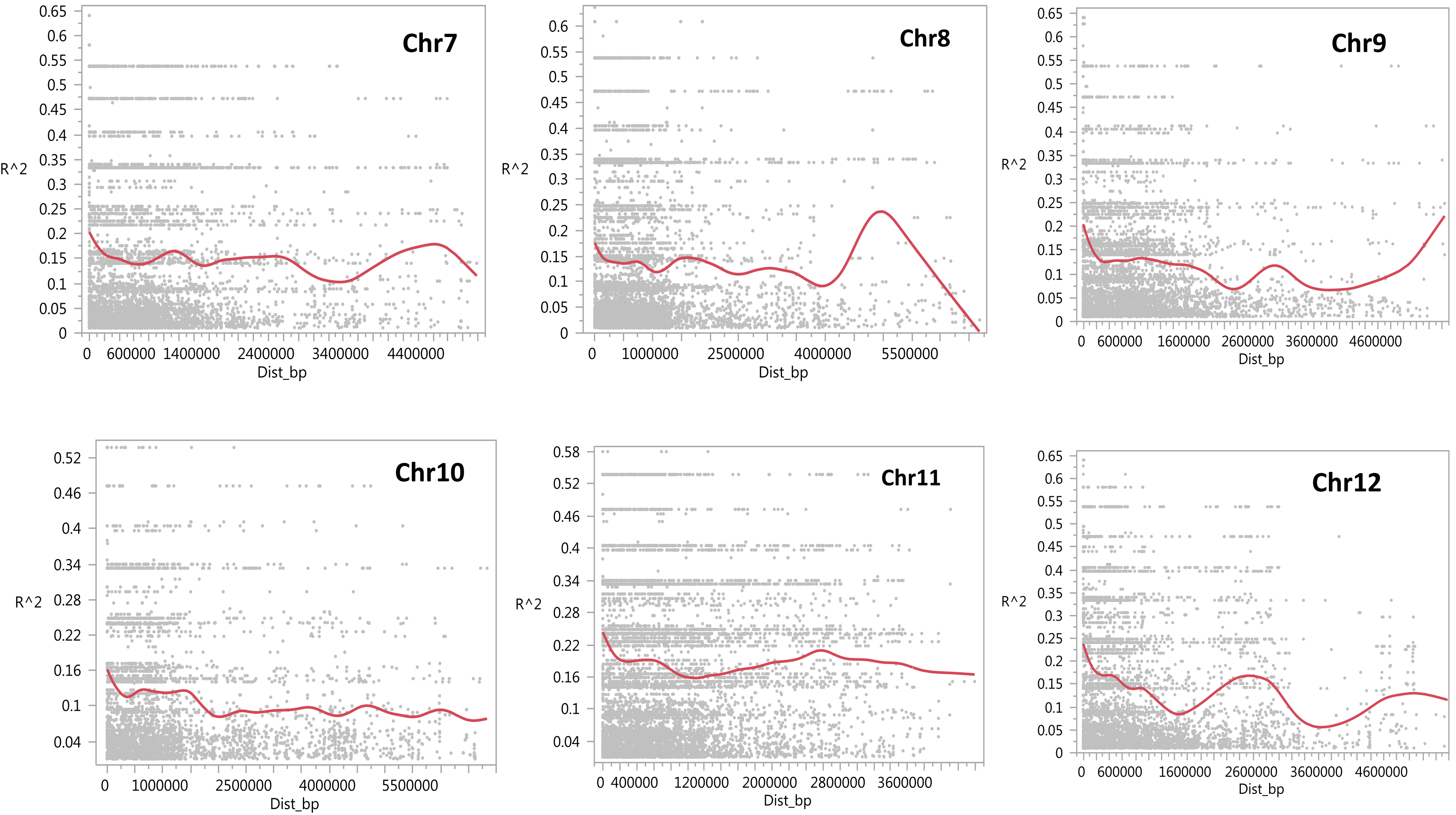

Supplementary Figure S5c: **LD decay plots by chromosomes within the *ssp. agrestis* group**. R^2 between intra-chromosomal marker pairs plotted against the physical distance between them.

a

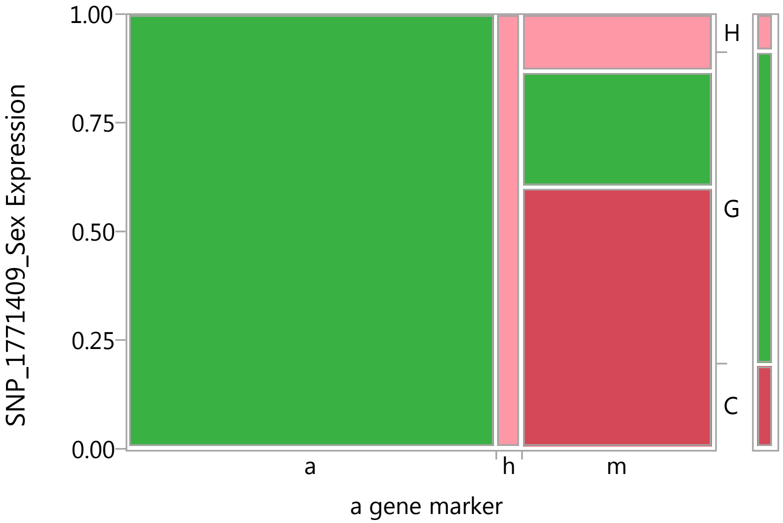

b

SNP_1771409_Sex Expression

a gene marker	Count / Total % / Col% / Row %	C	G	H	Total
a	Count	0	29	0	29
	Total %	0.00	63.04	0.00	63.04
	Col%	0.00	87.88	0.00	
	Row %	0.00	100.00	0.00	
h	Count	0	0	2	2
	Total %	0.00	0.00	4.35	4.35
	Col%	0.00	0.00	50.00	
	Row %	0.00	0.00	100.00	
m	Count	9	4	2	15
	Total %	19.57	8.70	4.35	32.61
	Col%	100.00	12.12	50.00	
	Row %	60.00	26.67	13.33	
Total	Count	9	33	4	46
	Total %	19.57	71.74	8.70	

Figure S6: **Validation of SNP_1771409 for flower sex-expression through alignment with PCR marker at the *CmACS-7* gene** (MELO3C015444, Boualem et al. 2008). (**a**) Mosaic plot of segregation of the two markers. (**b**) contingency table of segregation data

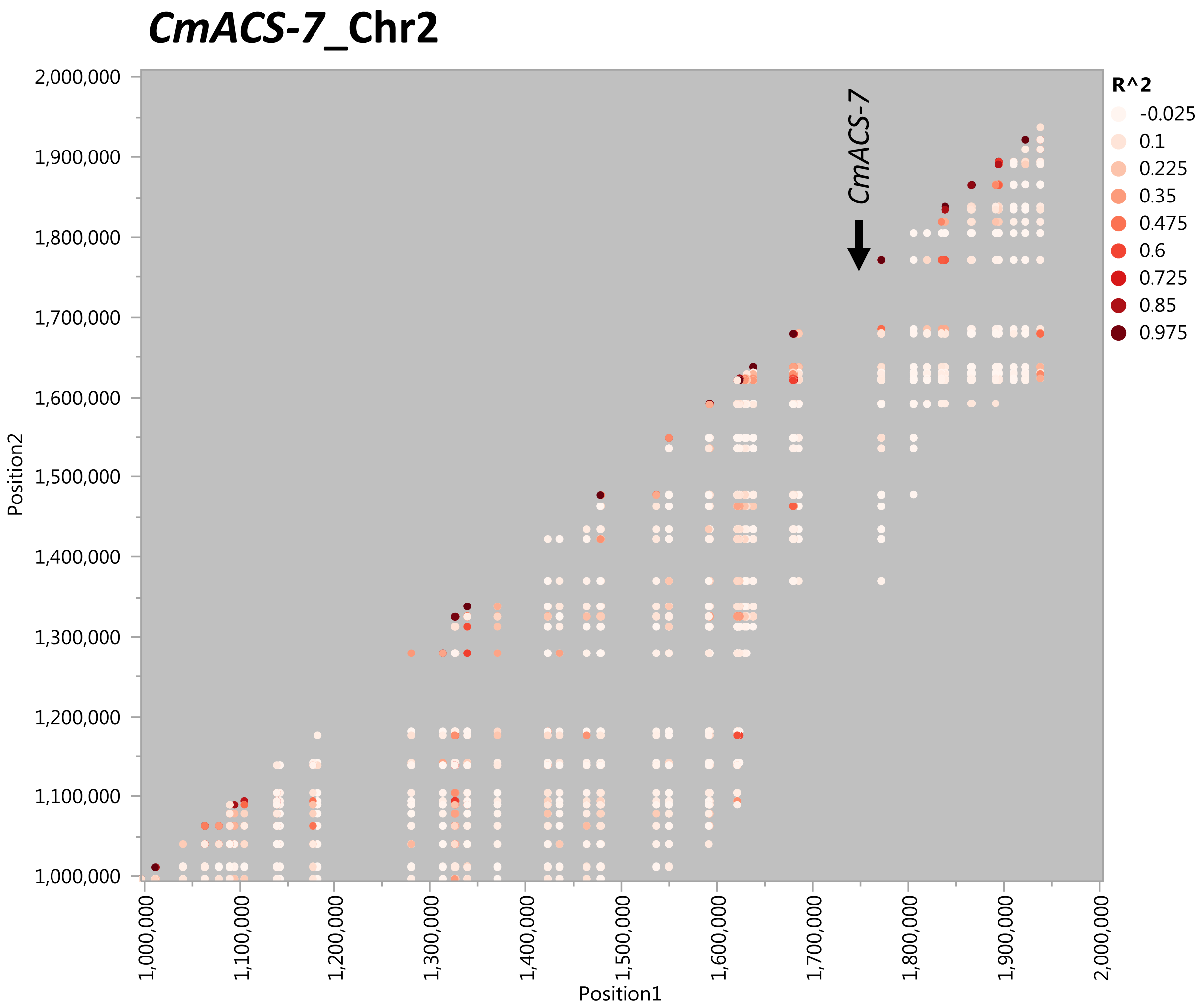

Supplementary Figure S7: LD distribution plot in a 1,000,000 bp interval surrounding the *CmACS-7* gene on chromosome 2.
LD is expressed as R2 and color-coded using white-to-Red scale.

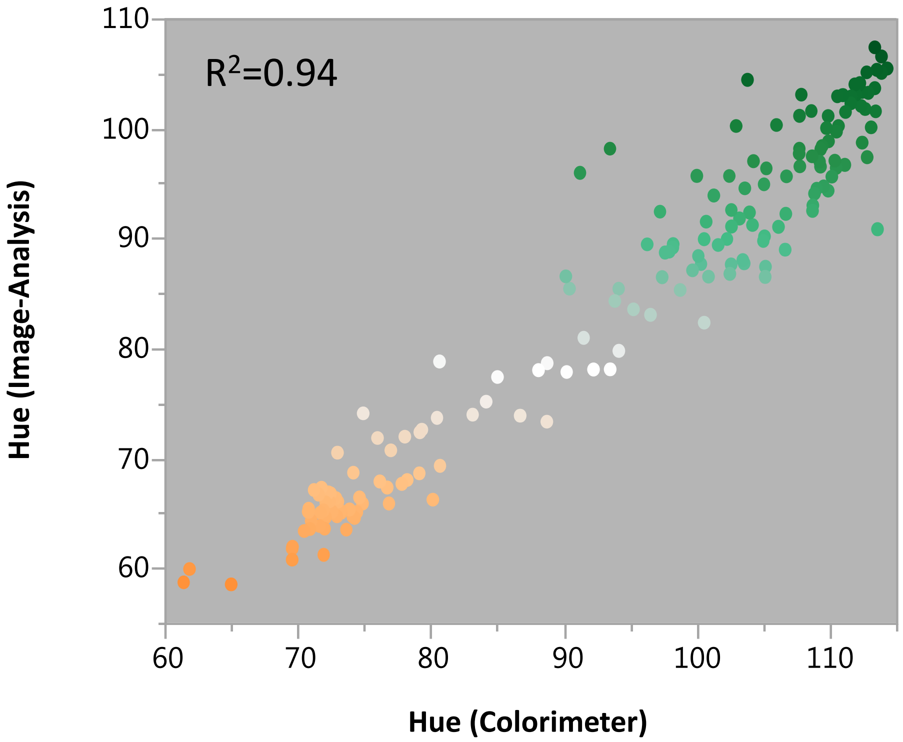

Supplementary Figure S8: **Correlation between fruit flesh hue, measured using colorimeter (X axis) and through fruit image analyses (Y axis).**

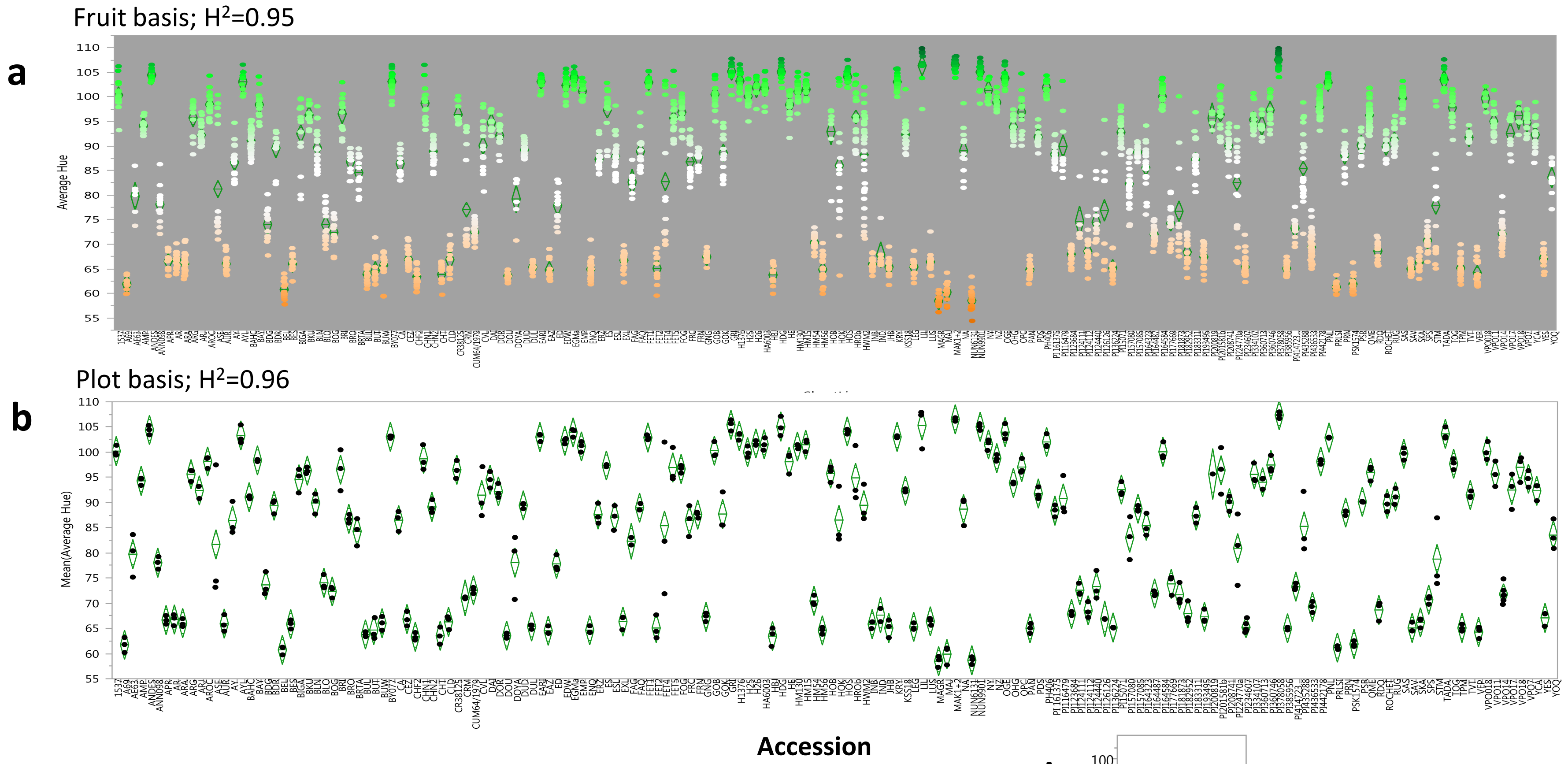

c

REML Variance Component Estimates						
**Random Effect**	**Var Ratio**	**Var Component**	**Std Error**	**95% Lower**	**95% Upper**	**Pct of Total**
Rep	0.005122	0.0613497	0.0741873	-0.084055	0.2067541	0.027
**Accession**	17.643397	211.32736	22.806405	166.62763	256.02709	94.610
Residual		11.977703	0.3211469	11.372371	12.632928	5.362
Total		223.36641	22.808432	184.62751	275.78996	100.000

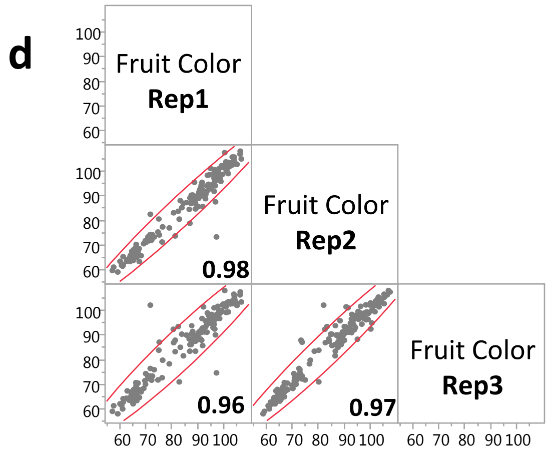

Supplementary Figure S9: Properties of flesh color variation . (a) one-way ANOVA at the fruit basis. (b) one-way ANOVA at the plot basis. (c) variation components table for the fruit basis ANOVA. (d) correlation between replications for plot means.

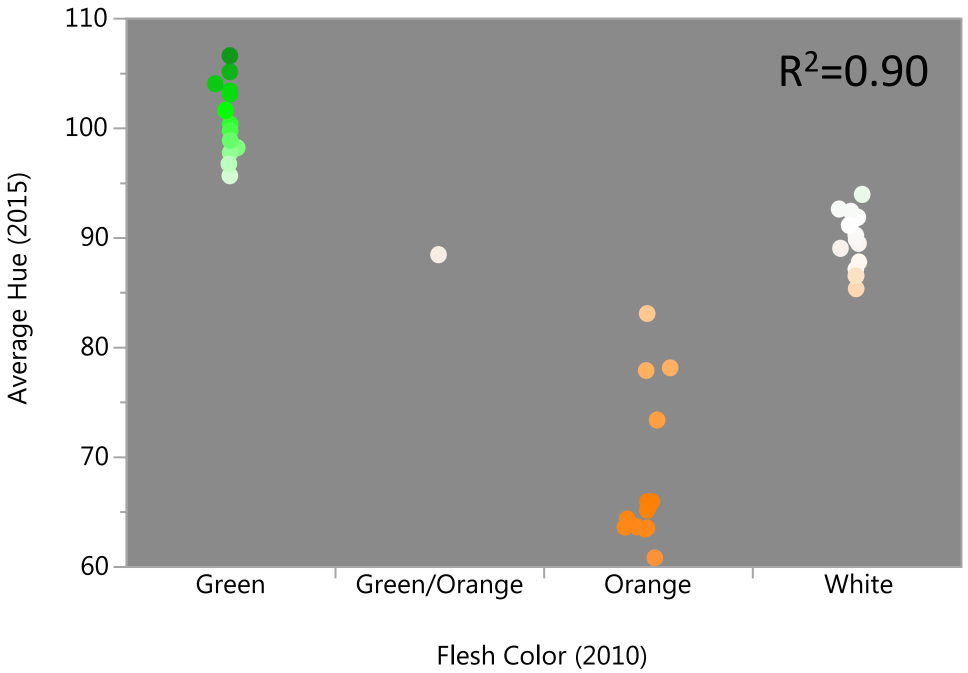

Supplementary Figure S10: **repeatability of flesh color in two different years across 43 diverse accessions.**
Each point represent accession mean. X axis are flesh color classes scores on the Summer 2010 experiment. On the Y axis are flesh color quantitative data collected from digital fruit images on Summer 2016 (see materials and methods).

a

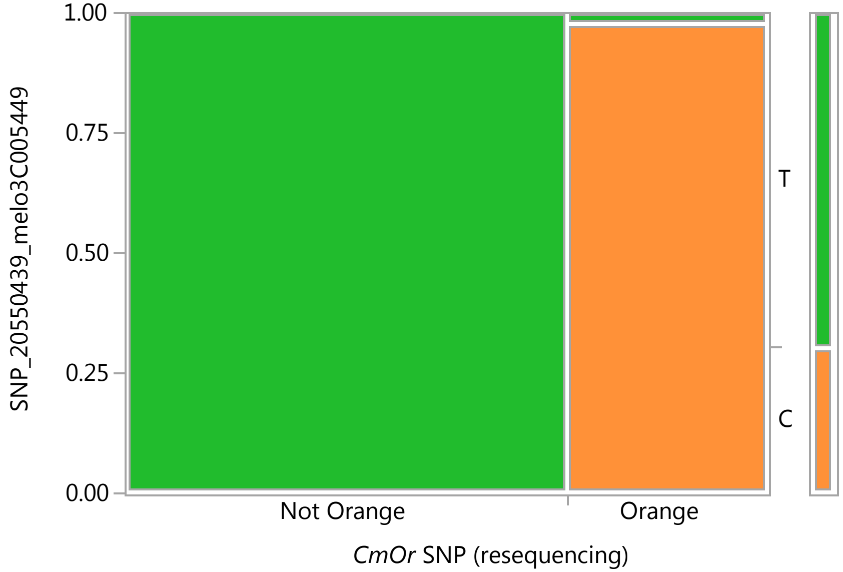

b

SNP_20550439_melo3C005449

CmOr SNP (resequencing)	Count<br>Total %<br>Col %<br>Row %	C	T	Total
Not Orange	Count	0	91	91
	Total %	0.00	68.94	68.94
	Col %	0.00	98.91	
	Row %	0.00	100.00	
Orange	Count	40	1	41
	Total %	30.30	0.76	31.06
	Col %	100.00	1.09	
	Row %	97.56	2.44	
Total	Count	40	92	132
	Total %	30.30	69.70	

Figure S11: **Validation of SNP_20550439 for flesh color through alignment with resequencing-based SNP at the *CmOr* gene** (melo3C005449, Tzuri et al. 2015). (**a**) Mosaic plot of segregation of the two markers. (**b**) contingency table of segregation data

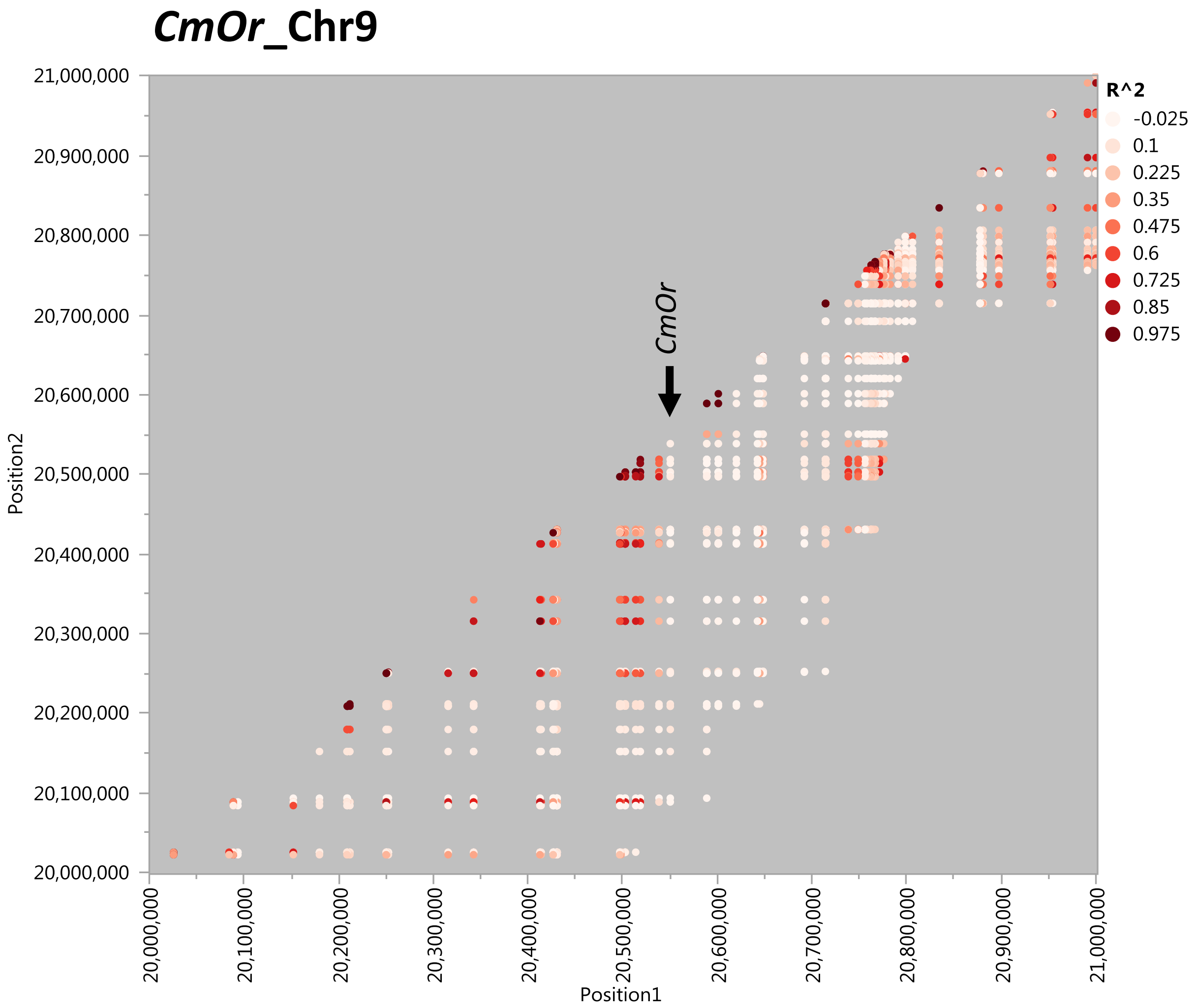

Supplementary Figure S12: **LD distribution plot in a 1,000,000 bp interval surrounding the *CmOr* gene on chromosome 9.**
LD is expressed as R2 and color-coded using white-to-Red scale.

a

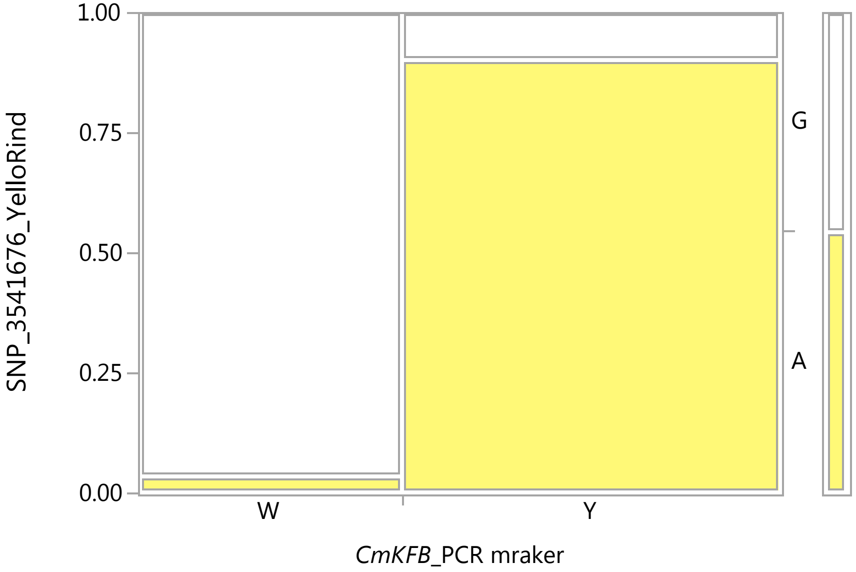

b

SNP_3541676_Yello Rind_

*CmKFB*_PCR Marker	Count<br>Total %<br>Col %<br>Row %	A	G	Total
W	Count	1	27	28
	Total %	1.47	39.71	41.18
	Col %	2.70	87.10	
	Row %	3.57	96.43	
Y	Count	36	4	40
	Total %	52.94	5.88	58.82
	Col %	97.30	12.90	
	Row %	90.00	10.00	
Total	Count	37	31	68
	Total %	54.41	45.59	

Figure S13: **Validation of SNP_3541676 for flesh color through alignment with PCR marker scoring an INDEL at the *CmKFB* gene** (MELO3C11980, Feder et al. 2015). (**a**) Mosaic plot of segregation of the two markers. W=White Allele, Y=Yellow allele. (**b**) contingency table of segregation data

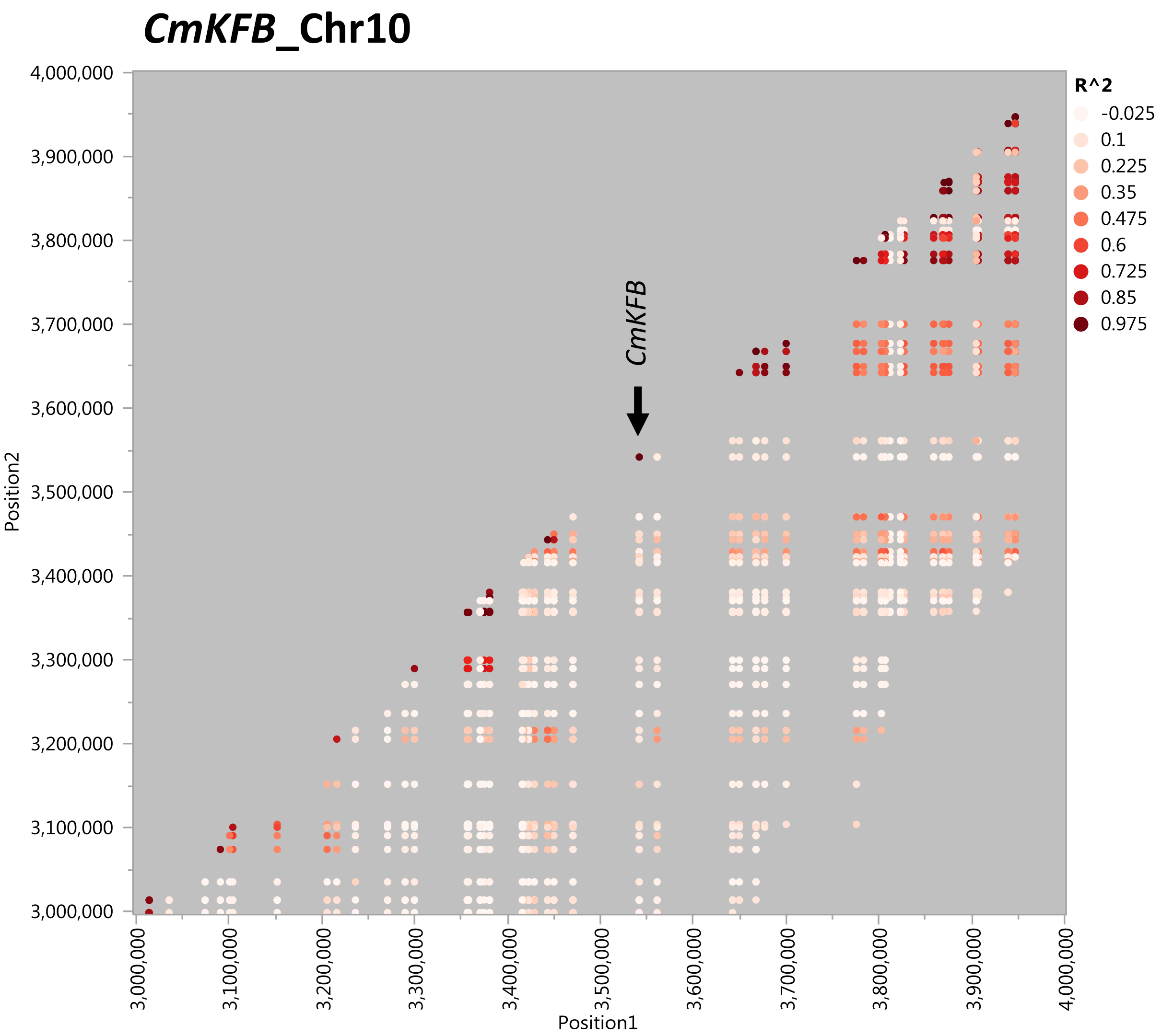

Supplementary Figure S14: LD distribution plot in a 1,000,000 bp interval surrounding the *CmKFB* gene on chromosome 10.
LD is expressed as R2 and color-coded using white-to-Red scale.

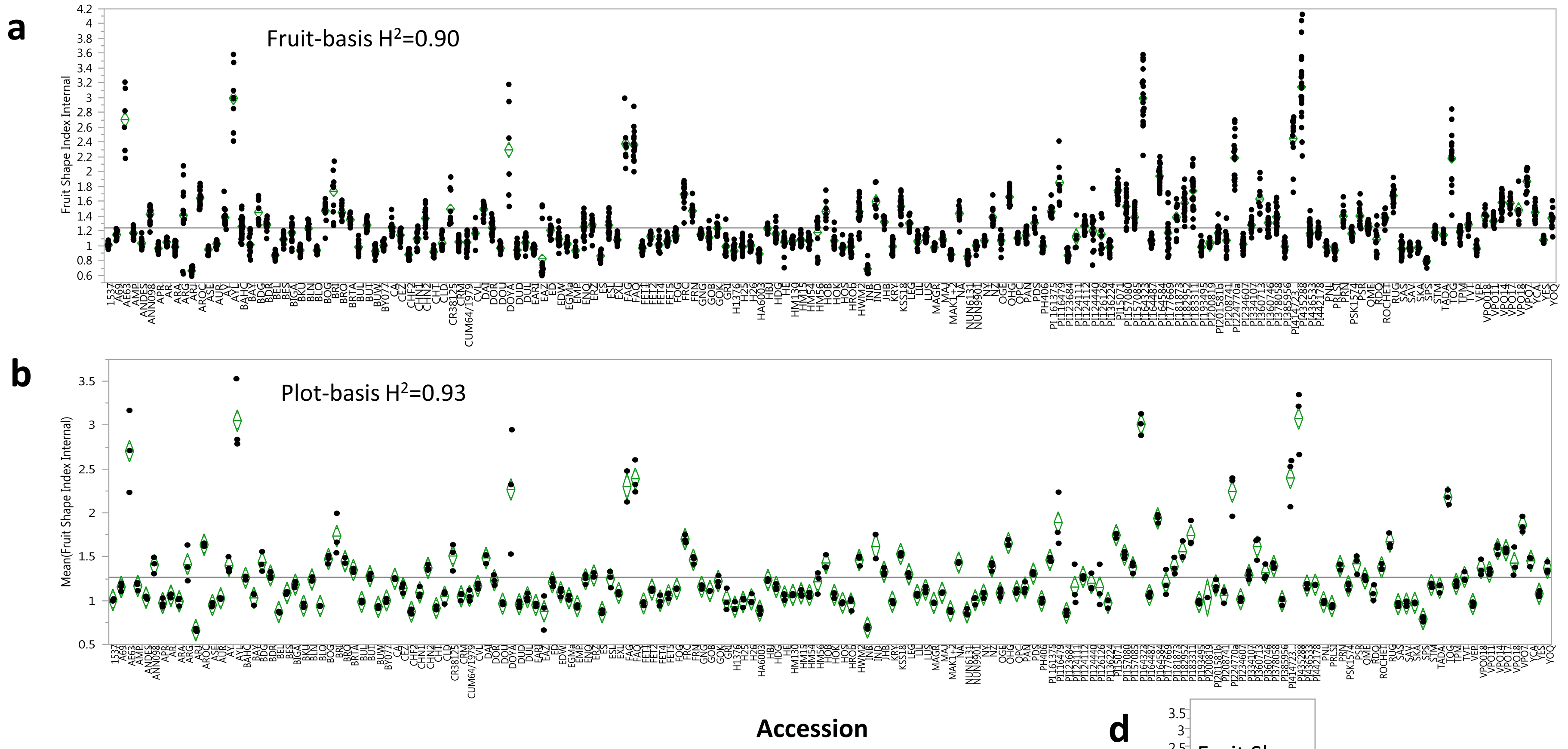

c

REML Variance Component Estimates						
**Random Effect**	**Var Ratio**	**Var Component**	**Std Error**	**95% Lower**	**95% Upper**	**Pct of Total**
Rep	0.0064533	0.0001207	0.0001404	-0.000155	0.0003959	0.065
**Accession**	8.8655302	0.1657838	0.0179868	0.1305303	0.2010372	**89.805**
Residual		0.0186998	0.0004998	0.0177576	0.0197194	10.130
Total		0.1846043	0.0179929	0.1538541	0.2256451	100.000

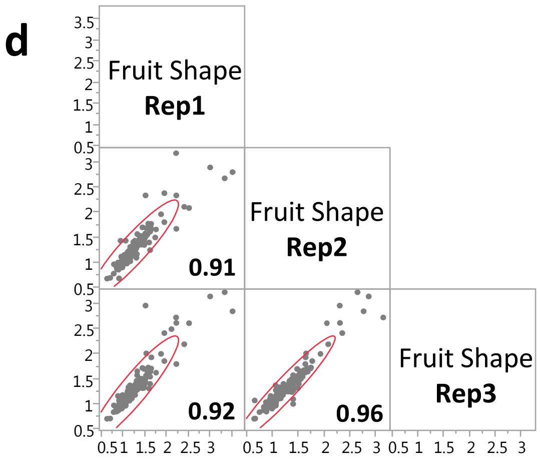

Supplementary Figure S15: **Properties of fruit shape variation .** (a) one-way ANOVA at the fruit basis. (b) one-way ANOVA at the plot basis. (c) variation components table for the fruit basis ANOVA. (d) correlation between replications for plot means.

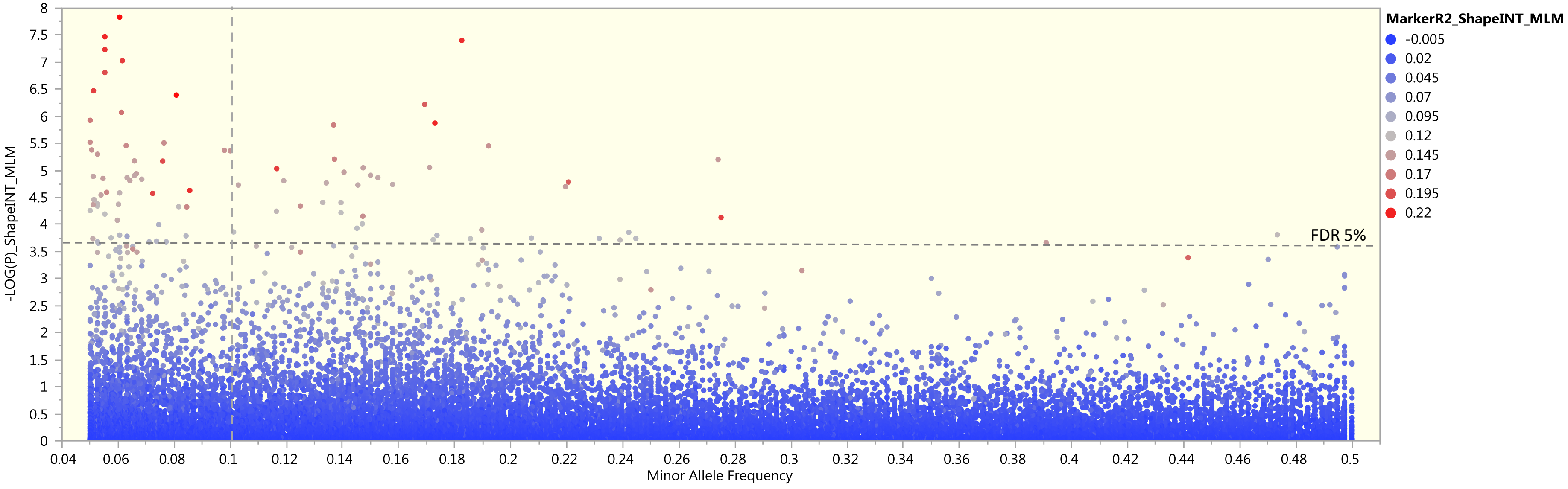

Supplementary Figure S16: Polymorphism effect size for fruit shape index and allele frequencies. SNP allele frequencies plotted against effects on fruit shape index (expressed as -Log P values). Effects are also colored based on SNP R^2.

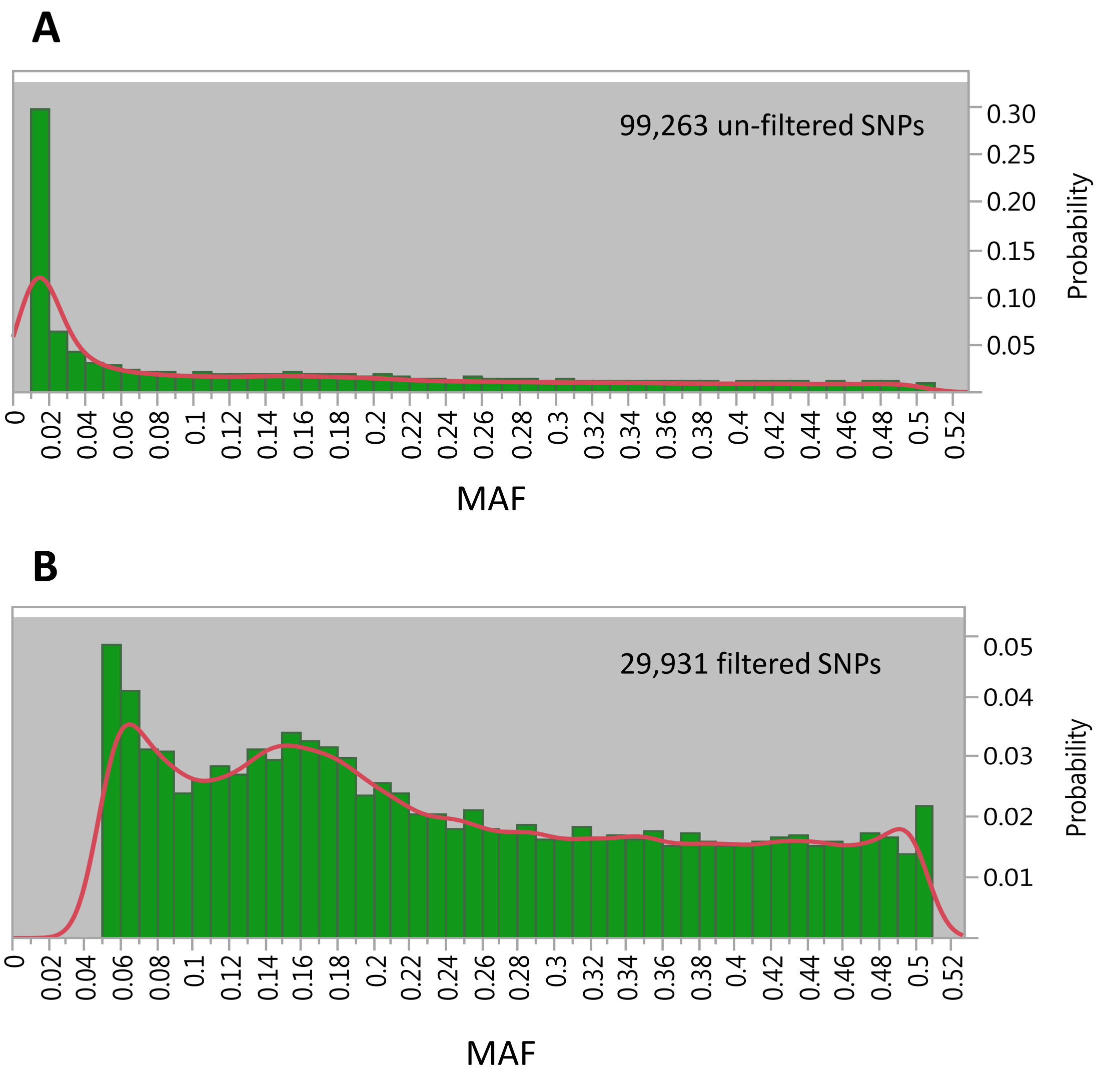

Supplementary Figure S17: **Frequency distribution of minor allele frequencies (MAF).** (A) Full set – 99,263 SNPs. (B) Filtered set - 23,931 SNPs.

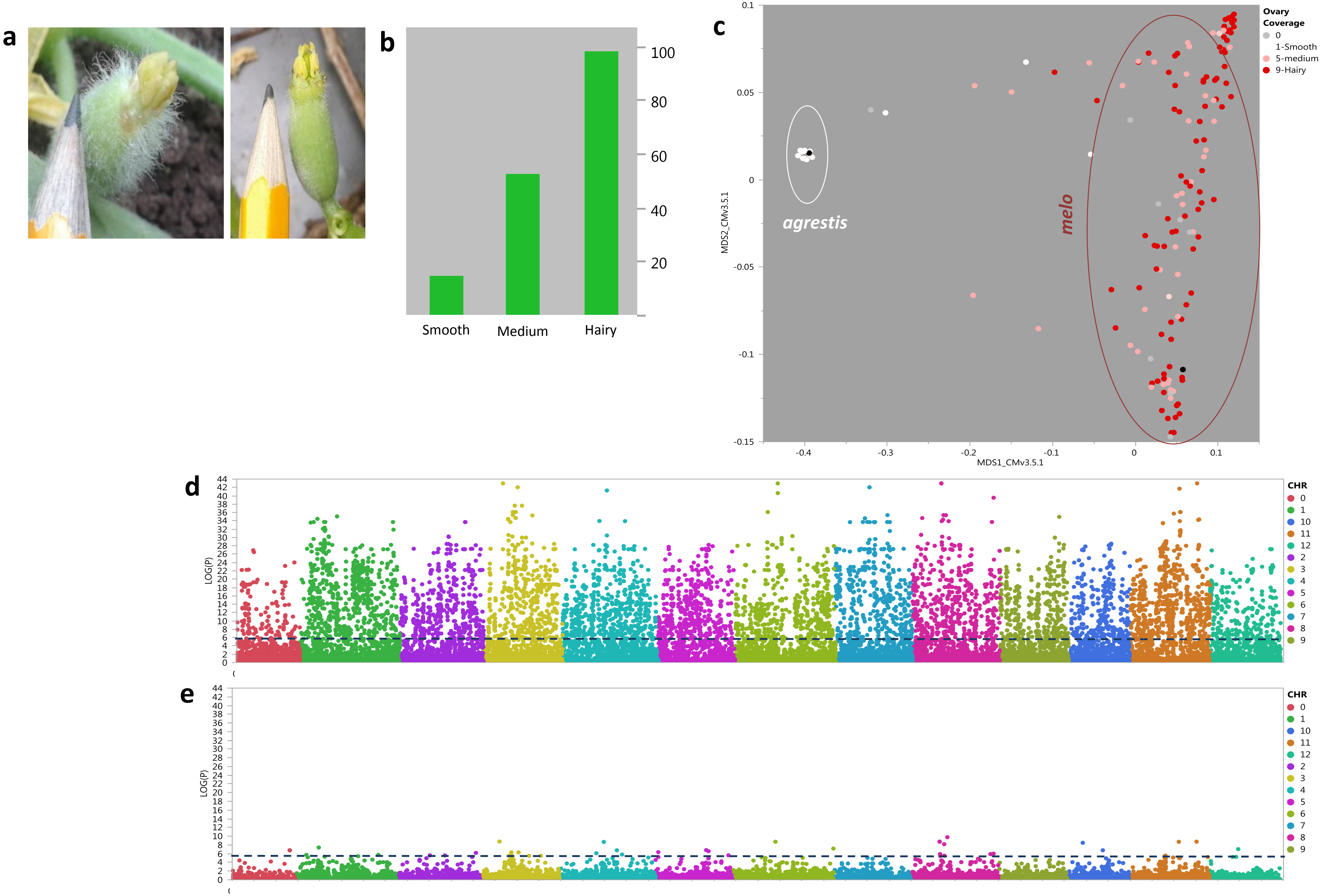

Supplementary Figure S18: **Characterization of ovary hairiness across the diversity panel** – (A) example of spreading hairs (left) and appressed hairs on ovaries at anthesis. (B) Frequency distribution of hairiness classes. (C) Ovary hairiness projected on genetic PCA plot. (D) Manhattan plots of GWA of ovary hairiness. GLM. (E) Manhattan plots of GWA of ovary hairiness. MLM.

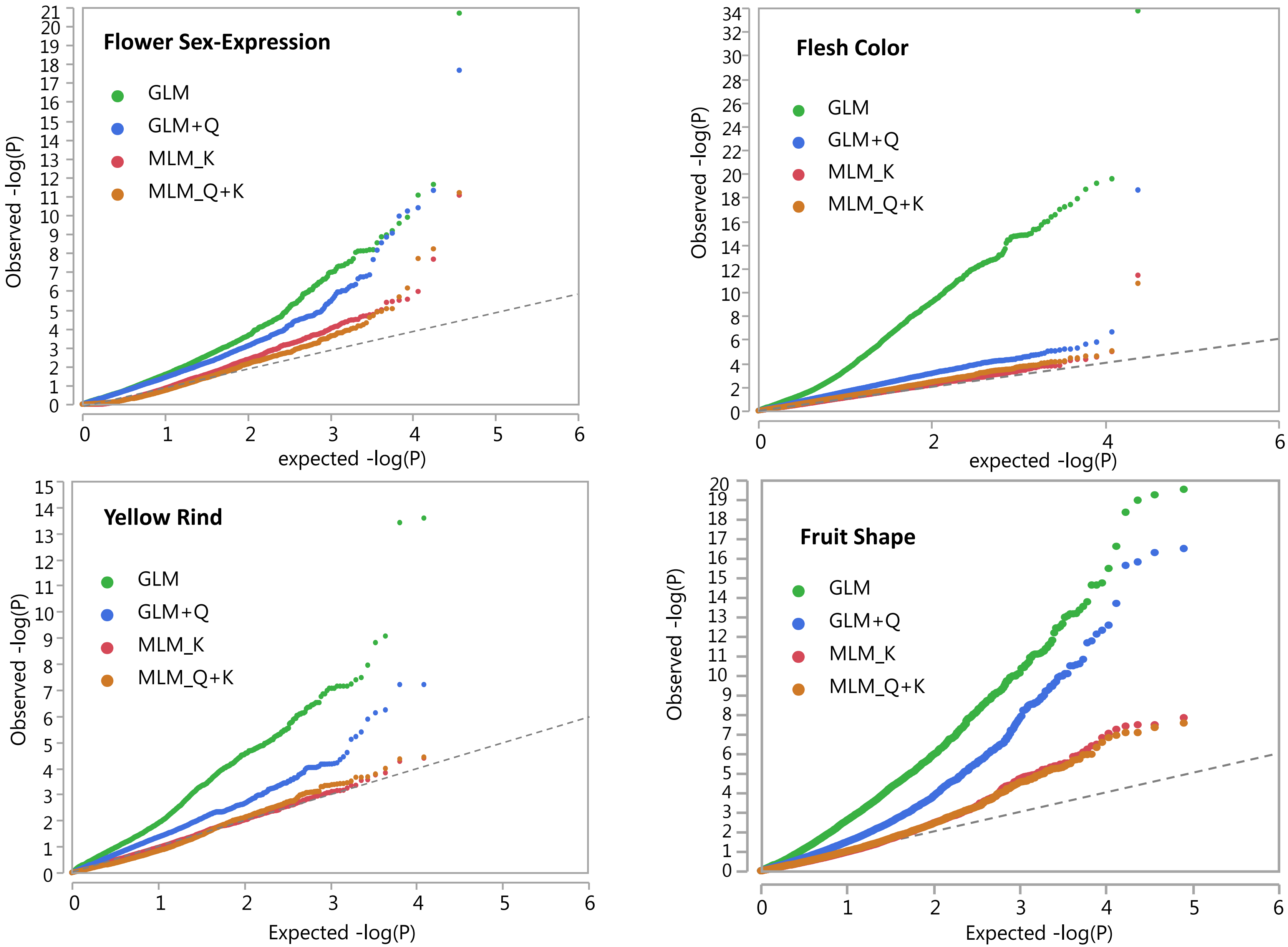

Supplementary Figure S19: **Quantile-quantile (Q-Q) plots comparing distribution of P values at the 4 statistical models used for GWA analyses**. The negative logarithm of the observed (y axis) and the expected (x axis) P value is plotted for each SNP (dot), and the gray dashed line indicates the null hypothesis of no true association.
